# Emotion regulation skills training as an adjunctive treatment to narrative exposure therapy for posttraumatic stress disorder (PTSD) in refugees: a pilot randomized controlled trial

**DOI:** 10.1080/20008066.2026.2648941

**Published:** 2026-04-08

**Authors:** Angela Nickerson, Marylene Cloitre, Frank Neuner, Meaghan O'Donnell, Richard A. Bryant, Belinda J. Liddell, Avalon Tissue, Stephanie Murphy, Casey Willoughby, Joel Hoffman, Yulisha Byrow, Shivani Uppal, Natalie Mastrogiovanni, Natalie Peach, Sarah Funnell, Vivian Mai, Marta Gurzeda, Jenny J.Y. Im, Gheed Al-Damook, Fariba Mozayani, Raghad Yacoub, Philippa Specker

**Affiliations:** a School of Psychology, UNSW Sydney, Sydney, NSW, Australia; b NYU Silver School of Social Work, New York, NY, USA; c Department of Psychology, University of Bielefeld, Bielefeld, Germany; d Phoenix Australia, University of Melbourne, Parkville, VIC, Australia; e School of Psychological Sciences, University of Newcastle, Callaghan, NSW, Australia; f School of Psychology, University of Sydney, Sydney, NSW, Australia; g Faculty of Medicine and Health, Sydney School of Medicine, The University of Sydney, Sydney, Australia

**Keywords:** Refugees, posttraumatic stress disorder, cognitive behvioural therapy, emotion regulation, exposure therapy, Refugiados, trastorno de estrés postraumático, terapia cognitivo conductual, regulación emocional

## Abstract

**Background:** Responses to evidence-based interventions for posttraumatic stress disorder (PTSD) in refugees vary considerably. Emotion regulation difficulties are associated with greater PTSD severity in refugees and thus represent a potential treatment target.

**Objective:** This study aimed to test the efficacy of Skills Training in Affective and Interpersonal Regulation for Refugees (STAIR-R) and Narrative Exposure Therapy (NET) or Supportive Problem-Solving (SPS) and NET. An exploratory aim was to examine relative efficacy in refugees with high and low levels of visa and family insecurity.

**Methods:** Seventy-one participants were randomly assigned to STAIR-R (6 sessions) + NET (7 sessions) or SPS (6 sessions) +NET (7 sessions). Assessments occurred at baseline, post-treatment, and 3-month follow-up, with additional self-report at mid-treatment. The primary outcome was clinician-assessed PTSD symptom severity (CAPS-5) and secondary outcomes included self-reported PTSD symptoms, depression symptoms, emotion regulation difficulties, difficulties in relationships and environmental quality of life.

**Results:** Intent-to-treat linear mixed models showed no significant between-group differences at mid-treatment, post-treatment, or follow-up. Both groups demonstrated significant improvements at 3-month follow-up in clinician-assessed (STAIR-R + NET, *g = −1.41*; SPS + NET, *g* = −1.54, *p* < .001) and self-reported (STAIR-R + NET, *g* = −0.49; SPS + NET, *g* = −0.44, *p* = .006) PTSD symptoms. Moderatorg analyses revealed that those with high insecurity (*n* = 16) obtained greater benefits in STAIR-R + NET from pre-treatment to follow-up on self-reported PTSD (*g* = 1.35), depression (*g* = 1.11), emotion regulation difficulties (*g* = 1.24), relationship difficulties (*g* = 1.12) and quality of life (*g* = −1.05).

**Conclusions:** While there were no overall between group-differences, refugees in both conditions showed reduced PTSD symptoms. There is preliminary evidence that those with high insecurity showed a better response to STAIR-R + NET than SPS + NET across several clinical outcomes, although the small sample size necessitates replication of these. These findings highlight the potential importance of tailored intervention approaches for refugees living in different environmental circumstances.

## HIGHLIGHTS

- Emotion regulation difficulties represent a promising treatment target for refugees with PTSD.
- A randomized controlled trial comparing Skills Training in Affective and Interpersonal Regulation for Refugees + Narrative Exposure Therapy (STAIR-R + NET) to Supportive Problem Solving Training + Narrative Exposure Therapy (SPS + NET) revealed no significant differences at 3-month follow-up, with both groups improving on PTSD symptoms.
- Moderator analyses provided preliminary evidence that refugees living with high insecurity (i.e. insecure visa or separated from all family) showed greater improvements following STAIR-R + NET than SPS + NET.

## Abstract (es)

**Antecedentes:** Las respuestas a las intervenciones basadas en la evidencia para el trastorno de estrés postraumático (TEPT) en refugiados varía considerablemente. Las dificultades en la regulación emocional se asocian con mayor gravedad del TEPT en refugiados y, por ende, representa un objetivo terapéutico potencial.

**Objetivo:** Este estudio tuvo como objetivo evaluar la eficacia del Entrenamiento de Habilidades en la Regulación Afectiva e Interpersonal para Refugiados (STAIR-R por sus siglas en inglés) y la Terapia de Exposición Narrativa (NET por sus siglas en inglés) o la Resolución de Problemas con Apoyo (SPS por sus siglas en ingles) y NET. Un objetivo exploratorio fue examinar la eficacia relativa en refugiados con altos y bajos niveles de inseguridad migratoria y familiar.

**Métodos:** Setenta y un participantes se asignaron en forma aleatoria al STAIR-R (6 sesiones) + NET (7 sesiones) o SPS (6 sesiones) + NET (7 sesiones). Las evaluaciones se hicieron al inicio, postratamiento y a los 3 meses de seguimiento, con autorreportes adicionales a la mitad del tratamiento. El resultado primario fue la gravedad de los síntomas de TEPT evaluada por el clínico (CAPS-5) y los resultados secundarios incluyeron síntomas de TEPT auto-informados, síntomas depresivos, dificultades en la regulación emocional, dificultades en las relaciones interpersonales y calidad de vida ambiental.

**Resultados:** Los modelos lineales mixtos por intención de tratar no mostraron diferencias significativas entre grupos a mitad del tratamiento, postratamiento o en el seguimiento. Ambos grupos demostraron mejoras significativas en el seguimiento de 3 meses en los síntomas de TEPT evaluados por el clínico (STAIR-R + NET, *g* = −1.41; 0.44, *p* = .006) y auto-informado (STAIR-R+NET, *g* = −0.49; SPS+NET, *g* = −0.44, *p* = .006) síntomas de TEPT. Los análisis de moderación revelaron que aquellos con alta inseguridad (*n* = 16) obtuvieron mayores beneficios en el STAIR-R + NET desde antes del tratamiento hasta el seguimiento en el TEPT auto-informado (*g* = 1.35), depresión (*g* = 1.11), dificultades en la regulación emocional (*g* = 1.24), dificultades en las relaciones (*g* = 1.12) y calidad de vida (*g* = 1.05).

**Conclusiones:** Si bien no hubo diferencias generales entre grupos, los refugiados mostraron en ambas condiciones una reducción de los síntomas de TEPT. Existe evidencia preliminar que aquellos con alta inseguridad mostraron una mejor respuesta a STAIR + NET que a SPS + NET en varios resultados clínicos, aunque el pequeño tamaño de la muestra necesita la replicación de estos resultados. Estos hallazgos destacan la importancia potencial de los enfoques de intervenciones personalizadas para los refugiados que viven en diferentes circunstancias ambientales.

Exposure to war, persecution and displacement confers heightened risk for posttraumatic stress disorder (PTSD) amongst refugees compared to the general population in high-income host countries (Fazel et al., 2005; Koenen et al., 2017; Patanè et al., 2022). This increased risk of psychopathology, alongside the growing number of forcibly displaced people worldwide (UNHCR, 2024), highlights the importance of evidence-based interventions to reduce psychological symptoms in refugees. Research and clinical guidelines point to the efficacy of trauma-focused interventions for treating PTSD in refugees (Kip et al., 2020; National Institute for Health and Care Excellence, 2018; Nosè et al., 2017; Phelps et al., 2021). There is considerable variability, however, in response to first-line treatments, with a large proportion of refugees failing to respond to evidence-based trauma-focused interventions (Haagen et al., 2017; Kip et al., 2020; Lambert & Alhassoon, 2015). There is also evidence that refugees show reduced response to trauma-focused interventions when compared to non-refugee trauma survivors (ter Heide & Smid, 2015). There is a need to better understand the factors that influence response to first-line psychological treatments amongst refugees to inform the refinement and targeting of existing interventions.

One potential pathway to improving refugee mental health lies in emotion regulation skills training. Stressors in the post-migration environment can give rise to negative emotions such as fear, sadness and anger (Li et al., 2016; Steel et al., 2006). Difficulties regulating these emotions have been found to mediate the cross-sectional relationship between ongoing stressors and greater psychological symptoms in refugees (Nickerson et al., 2015) and have been associated with subsequent increases in PTSD symptoms longitudinally (Specker et al., 2024). It is plausible that addressing difficulties regulating negative emotions arising from current stressors, could help improve daily functioning as well as facilitate symptom reduction in refugees receiving trauma-focused interventions. If this is the case, building skills in emotion regulation prior to engaging in trauma-focused therapy may facilitate increased treatment engagement and response.

Skills Training in Affective and Interpersonal Regulation (STAIR) is a short-term psychological intervention focusing on the development of emotion regulation and interpersonal skills (Cloitre et al., 2020). When combined with exposure therapy, STAIR has led to greater reductions in PTSD symptoms and improved treatment adherence amongst outpatient survivors of childhood sexual abuse, compared to supportive counselling combined with exposure therapy and STAIR combined with supportive counselling (Cloitre et al., 2010). Since this initial study, STAIR has been effectively implemented with civilian and military samples with PTSD (Cloitre et al., 2024a; Jain et al., 2020; MacIntosh et al., 2018). As this intervention specifically targets emotion regulation skills, it represents a promising treatment approach to be implemented in conjunction with trauma-focused treatments to reduce PTSD in refugees. In this study, we sought to examine whether the leading trauma-focused intervention for PTSD in refugees (Elbert et al., 2022; Kip et al., 2020; Nosè et al., 2017) could be enhanced by preceding this treatment with STAIR. We expected that augmenting NET with STAIR may improve treatment outcomes by targeting emotion regulation difficulties that maintain PTSD in refugees, and providing participants with skills they can use to manage both daily stressors and distress relating to confronting trauma memories (Nickerson et al., 2015; Specker et al., 2024).

In addition to determining whether STAIR shows overall effectiveness in reducing PTSD symptoms in refugees, it is important to investigate the differential impact of this approach for particular subgroups of refugees. This is consistent with calls for personalized medicine approaches to addressing psychological symptoms (Ozomaro et al., 2013). Refugees living in circumstances of heightened insecurity – such as those with insecure visas or who are separated from their families – show heightened risk for PTSD and other psychological disorders (Laban et al., 2008; Liddell et al., 2022; Momartin et al., 2006; Newnham et al., 2019; Nickerson et al., 2023). They are also subject to greater contextual stressors and ongoing threat (e.g. fear for family, fear of deportation) than those with secure visas and family members with them in the host country (Fogden et al., 2020; Liddell et al., 2022; Newnham et al., 2019; Nickerson et al., 2010, 2019; Steel et al., 2006). In these conditions, lack of control over one’s environment may negatively impact on the individual’s capacity and motivation to engage in past-oriented trauma-focused therapy (Semmlinger & Ehring, 2022). While research has found that refugees with insecure visa status respond to trauma-focused interventions (Haagen et al., 2017; Neuner et al., 2010; Stenmark et al., 2013; ter Heide & Smid, 2015), there is some evidence that higher levels of insecurity may hamper treatment retention and response (Djelantik et al., 2020). An outstanding question is how to most effectively target psychological symptoms amongst refugees living in contexts of high insecurity.

In this study, we evaluated whether a version of STAIR adapted for refugees (STAIR-R) would be effective in reducing PTSD symptoms (Tissue et al., 2023). The adapted intervention focused on building emotion regulation skills across three domains: the body (self-care), actions (behavioural activation) and the mind (positive coping statements). This intervention also focused on enhancing emotion regulation in an interpersonal context and building community connections. This adaptation was made due to the collectivistic self-construal of many of our refugee participants, in which social harmony and responsibilities may be prioritized over individual wellbeing (Liddell et al., 2024). In the current study, STAIR-R was delivered prior to NET, and compared to a control condition in which a client-led supportive problem-solving (SPS) intervention was delivered prior to NET. The SPS comparison condition was selected as (1) it maps onto treatment-as-usual for refugees, with many refugee-focused services using client-centred, non-directive supportive interventions that focus on current stressors (van Wyk & Schweitzer, 2014), (2) supportive counselling and problem-solving interventions are commonly employed to represent usual practice in randomized controlled trials evaluating treatments for PTSD in refugees (Bass et al., 2016; Neuner et al., 2004, 2008), and (3) the problem-solving focus of the comparison condition is in alignment with World Health Organization recommendations for the implementation of problem-solving approaches with refugees (Dawson et al., 2015). Accordingly, this study investigated whether an intervention targeting emotion regulation skills would lead to improved treatment outcomes compared to standard clinical practice when delivered prior to trauma-focussed therapy. Specifically, we hypothesized that STAIR-R followed by NET (STAIR-R + NET) would be more effective than SPS followed by NET (SPS + NET) in improving PTSD symptoms (primary outcome), depression symptoms, emotion dysregulation, relationship difficulties, and environmental quality of life (secondary outcomes). An exploratory aim of this study was to examine the moderating effect of insecurity on the efficacy of these interventions. Based on research linking environmental insecurity to emotion regulation difficulties in refugees (Nickerson et al., 2015), we hypothesized that the treatment effect would be moderated by insecurity status, such that refugees with high insecurity (defined as holding an insecure visa status or being separated from all immediate family) would benefit more from STAIR-R + NET than SPS + NET, compared to refugees with low insecurity.

## 1. Method

### 1.1. Study design

This study was a randomized parallel controlled trial. Participants were randomly assigned to STAIR-R + NET or SPS + NET on a 1:1 basis, stratified by visa insecurity.^1^ Treatment condition assignment was conducted by the project coordinator using a computerized system (REDCap) (Harris et al., 2009). Assessments were undertaken by trained psychologists and/or research assistants who were blind to treatment condition. The primary outcome was PTSD symptoms, measured by the Clinician Administered PTSD Scale (CAPS-5; Weathers et al., 2018) and the PTSD Checklist(PCL-5; Bovin et al., 2016). Assessments were conducted at pre-treatment, post-treatment, 3-month follow-up and 12-month follow-up (for which assessments are ongoing), with an additional self-report assessment between the STAIR/SPS and NET treatment phases. The primary time-point was the 3-month follow-up assessment.

### 1.2. Participants

Participants were recruited via referrals from community health and refugee treatment services throughout Australia. Participants were screened via telephone by psychologists or research assistants, with interpreter assistance. Inclusion criteria were (a) aged over 18 years, (b) refugee or asylum-seeker background, (c) fluent and literate in Arabic or Farsi, (d) met DSM-5 diagnostic criteria for PTSD, as determined by the Clinician Administered PTSD Scale (CAPS-5; Weathers et al., 2018), and (e) if receiving concurrent pharmacological treatment, were on a stable dose for one month prior to completing initial assessment. Exclusion criteria were (a) experiencing active suicidality (assessed using a suicide risk screener, where active suicidality was defined as suicidal intent in the past two weeks and/or suicide attempt in the past three months), (b) active psychosis or alcohol/substance dependence (assessed using the MINI International Neuropsychiatric Interview version 7.0.2, modules K [any psychotic disorder], I [alcohol use disorder] and J [substance use disorder (non-alcohol)]), or (c) moderate to severe brain injury (assessed using a traumatic brain injury screener, where moderate to severe brain injury was defined as experiencing a prior head injury(s) that resulted in hospitalization and/or loss of consciousness, and exhibiting clinically significant levels of common postconcussive symptoms such as headaches, dizziness, balance problems, memory problems).

Participants provided informed consent, and ethics approval was obtained from the UNSW Human Research Ethics Committee *(HC180551*). The authors assert that all procedures contributing to this work comply with the ethical standards of the relevant national and institutional committees on human experimentation and with the Helsinki Declaration of 1975, as revised in 2008. The study protocol was prospectively registered on the Australian and New Zealand Clinical Trials Registry (ACTRN12619000381189).

### 1.3. Procedures

#### 1.3.1 Treatment conditions

##### 1.3.1.1 STAIR-R + NET

The first phase of this condition was STAIR-R (Tissue et al., 2023), adapted from the original STAIR manual (Cloitre et al., 2020). This phase comprised six sessions, focused on (1) treatment rationale and calm breathing exercise, (2) psychoeducation on emotional responding, (3) physiological regulation strategies (e.g. sleep hygiene, diet, exercise), (4) behavioural regulation strategies (e.g. pleasant events scheduling, mastery and skill development), (5) cognitive regulation strategies (e.g. developing positive coping statements), and (6) review of emotion regulation skills and plans for future skill use. The second phase of treatment was NET (Schauer et al., 2011). This seven-session phase focused on the integration of traumatic events with positive and contextual life events to create a coherent autobiographical narrative (Schauer et al., 2011).

##### 1.3.1.2 SPS + NET

The first phase of this condition was six sessions of client-directed supportive problem-solving. We used an adapted version of a supportive counselling manual used in previous PTSD treatment studies (Bryant et al., 2003). In these sessions, therapists implemented supportive (positive regard, active listening, and empathetic reflection) and change (client-directed problem-solving) techniques to help participants better understand their practical problems and emotional responses to these, and to identify available resources to help them overcome these problems. This treatment phase was client-led, with clients focusing on whatever they identified as most challenging or salient for them in the past week. Discussion of past traumatic events, and the provision of emotion-focused coping strategies, was avoided. Phase two of treatment (NET) was identical across conditions.

### 1.4. Training, supervision, and fidelity assessment

The same therapists delivered both treatment conditions. Therapists (two masters-level and five doctoral-level clinical psychologists) received formal training in the treatment manuals via workshops from AN and FN, ongoing weekly supervision from AN and ad-hoc supervision as required from MC and FN. At the outset of the study, participants were seen face-to-face by the therapist and interpreter at an outpatient clinic in Sydney. Following the onset of the COVID-19 pandemic, participants were seen online via zoom.

All treatment sessions were digitally recorded and a random subset of sessions (10%; stratified across clinicians and treatment phases) was reviewed for treatment adherence. Tapes were rated by a research assistant, who was trained in the fidelity checklists for STAIR-R, SPS and NET, and supervised by PS, a doctoral-level clinical psychologist with expertise in all treatments. Approximately 10% of these tapes were double rated by PS, and inter-rater agreement was 100%. Therapist adherence to all treatments was high. The presence of required elements (62 treatment components) across all treatments was high (96.1% for STAIR-R, 90.0% for SPS and 98.7% for NET) and proscribed elements (e.g. exposure to trauma memories in SPS or STAIR, emotion regulation skills training in SPS) were successfully avoided in 100% of reviewed sessions. Overall treatment quality was also rated as high, with means of 5.89 (*SD* = 0.32) for STAIR-R, 5.88 (*SD* = 0.33) for SPS, and 5.92 (*SD* = 0.37) for NET (possible range: 0 = *unacceptable quality*, 6 = *high quality*).

### 1.5. Assessment

#### 1.5.1 Insecurity

Visa insecurity and family separation were assessed during the initial clinical interview and verified via self-report, measures (i.e. ‘What is your current visa status?’–Australian citizen or permanent protection visa, Temporary Protection Visa or Safe Haven Enterprise Visa, Bridging Visa, Expired Visa, No Visa; ‘What is your family status?’– no, some or all immediate family in Australia). Participants were categorized as high insecurity if they did not have Australian citizenship or a Permanent Protection visa and/or they were separated from all their immediate family.

#### 1.5.2 Clinician-administered measures

PTSD symptoms were assessed using the CAPS-5 (Weathers et al., 2018), a structured clinical interview that indexes the 20 PTSD symptoms detailed in the DSM-5 criteria. Each symptom is rated on a 5-point scale in terms of severity in the past month. The CAPS-5 showed good internal consistency at baseline, α = 0.80. This scale has been previously used with refugees in randomized controlled trials (Ertl et al., 2011; Stenmark et al., 2013; Ter Heide et al., 2016).

#### 1.5.3 Self-report

Self-reported PTSD symptoms were measured by the PCL-5 (Bovin et al., 2016), α = 0.93 at baseline. Depression symptoms were measured by the Beck Depression Inventory-II (Beck et al., 1988), α = 0.91 at baseline. Emotion dysregulation was indexed by two items from the International Trauma Questionnaire (ITQ): ‘When I am upset, it takes me a long time to calm down’ and ‘I feel numb or emotionally shut-down’ (Cloitre et al., 2018), ρ = 0.61 at baseline. Difficulties in relationships were indexed by two items from the ITQ: ‘I feel distant or cut-off from people’ and ‘I find it hard to stay emotionally close to people’, ρ = 0.66 at baseline. Environmental quality of life was indexed using the 8-item Environmental subscale of the World Health Organization Quality of Life Scale (WHOQOL-BREF; Noerholm et al., 2004; WHOQOL Group, 1998), α = 0.80 at baseline.

### 1.6. Data analysis

We based the projected sample size on a prior study comparing STAIR + Prolonged Exposure to Supportive Counselling + Prolonged Exposure, which found a between-groups pre- to post-treatment effect size of 0.73 on the CAPS (Cloitre et al., 2010). We conservatively expected an effect size of at least *d* = 0.65 between conditions. To detect an effect size of 0.65, with 80% power at a = 0.05, we calculated a necessary sample size of 64 participants per condition (a total of 128 participants). Due to disruptions in recruitment related to COVID-19 and funding limitations, our final sample size was 71 participants, meaning this study was under-powered. To investigate statistical sensitivity in light of our sample size, we calculated the minimum detectable effect size given our sample size. With the sample size of *n* = 71, a two-tailed α = 0.05 with 80% power would detect a moderate-to-large standardized between group difference. Given our observed between-group difference in the primary outcome at the three-month follow-up was small (*g* = −0.19), this study was underpowered to detect small effect.

All analyses were undertaken in R version 4.4.2 (R Core Team, 2021). *T*-tests and chi-square analyses were used to investigate differences between participants across treatment groups and between those who completed the 3-month follow-up assessment and those who did not. We used intent-to-treat linear mixed models with maximum likelihood estimation methods. For all primary and secondary outcomes, we modelled linear time from pre-treatment to post-treatment and 3-month follow-up (for clinician-administered measures) and from pre-treatment to mid-treatment, post-treatment and 3-month follow-up (for self-report measures). We included a random intercept, fixed effects (time of assessment and treatment condition) and their interactions to investigate relative change in symptoms across treatment conditions over time. Fixed effects parameters were evaluated with the Wald test (*t*-test, *p* < .01, two-sided) and 95% confidence intervals.

For our exploratory aim, we investigated symptom change amongst those with high (i.e. insecure visa and/or separated from all immediate family) vs low insecurity status (i.e. secure visa and at least some immediate family in Australia). Small sample sizes precluded investigation of visa status and family separation separately. For these analyses, we included an additional fixed effect (insecurity status) into the abovementioned model, as well as interactions to represent time × insecurity status, condition × insecurity status, and time × condition × insecurity status. We also included centred baseline scores to control for baseline differences in symptoms across insecurity status groups. As we were particularly interested in symptom change across conditions for those in the high insecurity status group, we conducted mean difference testing for this group at each time-point across treatment conditions. Given the small sample size, we used Hedges’ *g* to determine effect sizes of between-group differences for refugees in the high insecurity status group in the STAIR + NET condition vs the SPS + NET condition (Cumming, 2011).

Data, study materials, code and the intervention manual are available from the first author at reasonable request.

## 2. Results

Eligibility assessments of participants (*n* = 108) were conducted between 8 April 2019 and 6 November 2023. Seventy-one participants were randomly assigned to STAIR-R + NET (*n* = 35) or SPS + NET (*n* = 36). There were no differences between conditions on baseline characteristics (see Table 1). There were no differences between those who completed 3-month follow-up and those who did not on baseline characteristics (see Supplementary Table A). See Supplementary Figure A for participant flow through the trial. Overall, 54 participants (76.1%) completed all 13 sessions of therapy, with no differences across conditions.

**Table 1.** Participant characteristics.

	Overall sample *N* = 71	STAIR-R + NET *n* = 35	SPS + NET *n* = 36	*t* or *χ^2^*
Age, years	46.79 (11.28)	45.25 (11.94)	48.28 (10.54)	*t*(69) = 1.29, *p* = .260
Sex				
Female	47 (66.2%)	26 (74.3%)	21 (58.3%)	*χ^2^*(1) = 2.02, *p* = .155
Male	24 (33.8%)	9 (25.7%)	15 (41.7%)	
PTE Exposure	11.82 (4.35)	11.17 (4.66)	12.57 (3.91)	*t*(63) = 1.30, *p* = .200
Time in Australia	4.26 (4.75)	3.90 (3.20)	4.61 (5.91)	*t*(69) = 0.40, *p* = .530
Insecurity status				
High insecurity status	16 (23.9%)	n = 7 (21.2%)	n = 9 (26.5%)	*χ^2^*(1) = 0.26, *p* = .614
Low insecurity status	51 (76.1%)	n = 26 (78.8%)	n = 25 (73.5%)	
Language				
Arabic	66 (93.0%)	34 (97.1%)	32 (88.9%)	*χ^2^*(2) = 2.38, *p* = .304
Farsi	2 (2.8%)	0 (0.0%)	2 (5.6%)	
English	3 (4.2%)	1 (2.9%)	2 (5.6%)	
Medication				
Yes	23 (46.0%)	12 (52.5%)	11 (40.7%)	*χ^2^*(1) = 0.65, *p* = .419
No	27 (54.0%)	11 (47.8%)	16 (59.3%)	
Therapy with interpreter				
Yes	69 (97.2%)	34 (97.1%)	35 (97.2%)	*χ^2^*(1) < 0.001, *p* = .984
No	2 (2.8%)	1 (2.9%)	1 (2.8%)	
Therapy modality				
Face-to-face only	14 (19.7%)	8 (22.9%)	6 (16.7%)	*χ^2^*(1) = 0.43, *p* = .512
Online or hybrid	57 (80.3%)	27 (77.1%)	30 (83.3%)	
Mean number of sessions attended	11.27 (3.47)	11.51 (3.26)	11.03 (3.70)	*t*(69) = 0.59, *p* = .559
Current diagnosis of major depressive disorder	49 (69.0%)	26 (74.3%)	23 (69.7%)	*χ^2^*(1) = 0.18, *p* = .673
PTSD symptoms (clinician-administered)	39.41 (9.27)	40.00 (9.68)	38.80 (8.92)	*t*(69) = 0.54, *p* = .295
PTSD symptoms (self-report)	50.36 (12.73)	48.63 (18.92)	52.00 (13.82)	*t*(64) = 0.83, *p* = .205
Depression symptoms	30.23 (12.73)	31.50 (14.06)	29.03 (11.42)	*t*(64) = −0.79, *p* = .435
Emotion dysregulation	4.89 (2.05)	5.24 (2.23)	4.56 (1.85)	*t*(64) = 1.30, *p* = .199
Relationship difficulties	4.57 (2.25)	4.90 (2.44)	4.28 (2.05)	*t*(56) = 1.07, *p* = .289
Environmental quality of life	10.81 (2.61)	10.87 (2.81)	10.75 (2.44)	*t*(63) = −0.19, *p* = .853

SD: standard deviation; PTE: potentially traumatic events; PTSD: posttraumatic stress disorder.

### 2.1. Primary outcome (Table 2, Supplementary Tables B and C)

Means, standard deviations and ranges for all variables are presented in Supplementary Tables B and C, alongside within-groups effect sizes. There was a significant effect of time from pre- to post-treatment (B = −15.50, SE = 2.13, *p* < .001) and follow-up (B = −14.50, SE = 2.13, *p* < .001), indicating that participants showed improved PTSD symptoms over time. There was no time x condition interaction for clinician-administered or self-reported PTSD symptoms. Within-group effect sizes for PTSD symptoms were as follows: from pre- to post-treatment *g* = −1.61 for STAIR-R + NET and *g* = −1.64 for SPS + NET; from pre-treatment to follow-up *g* = −1.41 for STAIR-R + NET and −1.54 for SPS-NET.

**Table 2.** Summary results of mixed models analysis investigating differential change in symptoms over time according treatment condition.

	Descriptive statistics		Mixed model analysis		
	STAIR-R + NET (*n* = 35) Estimated Marginal Mean	SPS + NET (*n* = 36) Estimated Marginal Mean	LS mean difference STAIR-R + NET vs SPS + NET	*p* value	Hedges' *g* (95% CI) STAIR-R + NET vs SPS + NET
CAPS score					
Baseline	38.80	40.11	1.31	.640	0.11 (−0.35 to 0.57)
Post-treatment	23.65	24.61	0.97	.746	0.08 (−0.38 to 0.54)
3-month follow-up	28.05	25.61	−2.44	.417	−0.19 (−0.65 to 0.27)
PCL score					
Baseline	48.85	52.02	3.17	.463	0.17 (−0.29 to 0.63)
Mid-treatment	44.75	44.98	0.23	.958	0.01 (−0.45 to 0.47)
Post-treatment	41.38	40.77	−0.60	.893	−0.03 (−0.49 to 0.43)
3-month follow-up	40.70	44.67	3.97	.391	0.20 (−0.29 to 0.66)
BDI score					
Baseline	31.76	29.19	−2.57	.457	−0.18 (−0.64 to 0.29)
Mid-treatment	29.65	24.99	−4.66	.198	−0.31 (−0.77 to 0.16)
Post-treatment	27.94	25.68	−2.26	.538	−0.15 (−0.61 to 0.32)
3-month follow-up	27.26	27.09	−0.17	.964	−0.01 (−0.47 to 0.45)
ITQ-Emotion Dysregulation score					
Baseline	5.27	4.57	−0.70	.212	−0.03 (−0.76 to 0.17)
Mid-treatment	4.68	3.91	−0.78	.190	−0.31 (−0.77 to 0.15)
Post-treatment	4.02	3.85	−0.16	.786	−0.06 (−0.52 to 0.40)
3-month follow-up	4.02	3.96	−0.06	.927	−0.02 (−0.48 to 0.44)
ITQ-Difficulties in Relationships score					
Baseline	5.03	4.26	−0.77	.206	−0.30 (−0.76 to 0.16)
Mid-treatment	4.32	3.85	−0.47	.455	−0.18 (−0.64 to 0.28)
Post-treatment	3.84	3.70	−0.15	.821	−0.05 (−0.51 to 0.41)
3-month follow-up	4.19	4.12	−0.08	.909	−0.03 (−0.49 to 0.43)
WHOQOL Environmental Domain score					
Baseline	10.86	10.64	−0.22	.737	−0.08 (−0.54 to 0.38)
Mid-treatment	11.55	10.97	−0.58	.396	−0.20 (−0.66 to 0.26)
Post-treatment	11.38	11.70	0.32	.642	0.11 (−0.35 to 0.57)
3-month follow-up	11.53	11.39	−0.14	.840	−0.05 (−0.51 to 0.41)

LS: Least squares; CAPS: Clinician Administered PTSD Scale; PCL: PTSD Checklist for DSM-5; BDI: Beck Depression Inventory; ITQ: International Trauma Questionnaire; WHOQOL: World Health Organization Quality of Life Scale.

### 2.2. Secondary outcomes (Table 2, Supplementary Table B)

There was a significant effect of time from pre- to mid- (B = −7.04, SE = 2.52, *p* = .006) and post-treatment (B = −11.25, SE = 2.57, *p* < .001) and follow-up (B = −7.36, SE = 2.64, *p* = .006) for PTSD symptoms, and from pre- to post-treatment for environmental quality of life (B = 1.06, SE = 0.39, *p* = .007). There was no time x condition interaction for depression, emotion dysregulation, relationship difficulties or environmental quality of life. Within-group effect sizes for PTSD symptoms were: from pre- to mid-treatment g = −0.25 for STAIR-R + NET and g = −0.42 for SPS + NET; from pre- to post-treatment g = −0.45 for STAIR-R + NET and −0.68 for SPS-NET; from pre-treatment to follow-up g = −0.49 for STAIR-R + NET and g = −0.44 for SPS + NET. Within-group effect sizes from pre- to post-treatment for environmental quality of life were g = 0.20 for STAIR_R + NET and *g* = 0.40 for SPS + NET.

### 2.3. Exploratory moderator analyses

Demographic details of those with high insecurity status (i.e. holding temporary visa or separated from all family) and low insecurity status are presented in Supplementary Table D. Participants with high insecurity status exhibited greater PTSD and depression symptoms at baseline, but did not differ from those in low insecurity in number of sessions completed. Means, standard deviations and ranges of variables across conditions and groups are presented in Supplementary Table E.

#### 2.3.1 Primary outcome (Table 3, Supplementary Table F)

**Figure 1.**
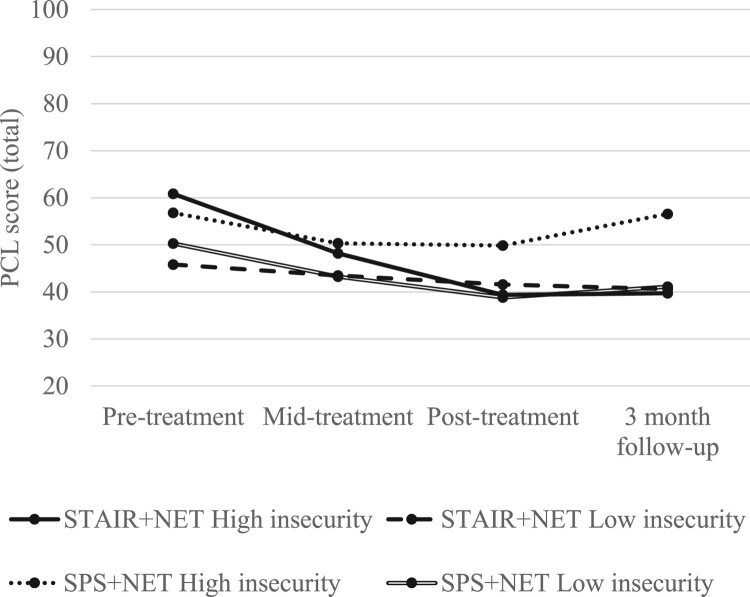
Differential changes in self-reported PTSD symptoms over time across treatment condition and insecurity status group. *NB Means displayed in figures did not control for baseline symptom severity to provide a more accurate representation of participant symptoms over the course of treatment. PTSD: posttraumatic stress disorder; PCL: Posttraumatic Stress Disorder Symptom Checklist.

On clinician-administered PTSD symptoms, there was no significant time *×* condition *×* insecurity group interaction. On self-reported PTSD symptoms, there was a significant time *×* condition *×* insecurity group interaction for PCL-5 scores from pre-treatment to follow-up, indicating that participants with high insecurity who were in the STAIR-R + NET condition showed greater decreases in PTSD symptoms than those in the SPS + NET condition (between-groups effect size [*g*] = 1.35, see Figure 1).

**Table 3.** Summary results of mixed models analysis investigating differential change in symptoms over time according to insecurity status and treatment condition.

	Descriptive statistics				Mixed model analysis		
	STAIR+ NET High insecurity (*n* = 7) Estimated Marginal Mean	STAIR+ NET Low insecurity (*n* = 26) Estimated Marginal Mean	SPS + NET High insecurity (*n* = 9) Estimated Marginal Mean	SPS + NET Low insecurity (*n* = 25) Estimated Marginal Mean	LS mean difference High insecurity STAIR + NET vs SPS + NET	*p* value	Hedges' *g* (95% CI) High insecurity STAIR + NET vs SPS + NET
CAPS score							
Baseline	39.36	38.95	40.95	38.78	1.59	.748	0.16 (−0.83 to 1.15)
Post-treatment	27.73	23.53	19.23	25.10	−8.50	.151	−0.73 (−1.86 to 0.39)
3-month follow-up	29.90	29.43	26.56	24.52	−3.34	.595	−0.26 (−1.26 to 0.73)
PCL score							
Baseline	51.18	48.84	50.55	49.54	−0.63	.921	−0.05 (−1.04 to 0.94)
Mid-treatment	38.51	46.56	44.22	42.88	5.71	.388	0.43 (−0.57 to 1.43)
Post-treatment	31.21	44.71	44.42	36.31	13.22	.074	0.93 (−0.12 to 1.99)
3-month follow-up	30.12	43.65	50.79	41.29	20.67	.015	1.35 (0.23 to 2.47)
BDI score							
Baseline	32.27	30.36	31.48	29.90	−0.80	.872	−0.08 (−1.07 to 0.91)
Mid-treatment	24.44	29.53	25.18	27.29	0.74	.884	0.07 (−0.92 to 1.06)
Post-treatment	24.39	27.60	30.52	24.89	6.13	.270	0.55 (−0.46 to 1.57)
3-month follow-up	25.48	26.23	38.88	24.95	13.40	.037	1.11 (0.04 to 2.19)
ITQ – Emotion Dysregulation score							
Baseline	6.50	5.07	4.67	4.56	−1.83	.183	−0.69 (−1.65 to 0.28)
Mid-treatment	3.77	4.80	4.26	3.79	1.95	.100	0.89 (−0.15 to 1.93)
Post-treatment	1.140	4.00	5.07	3.56	3.93	.008	1.57 (0.41 to 2.77)
3-month follow-up	2.14	3.82	5.32	3.82	3.18	.028	1.24 (0.14 to 2.34)
ITQ – Difficulties in Relationships score							
Baseline	4.95	4.55	4.43	4.46	−0.51	.613	−0.24 (−1.19 to 0.69)
Mid-treatment	3.29	3.98	4.52	3.71	1.23	.276	0.55 (−0.40 to 1.50)
Post-treatment	2.93	3.43	5.12	3.45	2.19	.093	0.86 (−0.12 to 1.84)
3-month follow-up	3.43	3.69	6.36	3.73	2.93	.027	1.12 (0.11 to 2.13)
WHOQOL Environmental Domain score							
Baseline	10.60	10.87	10.80	10.79	0.20	.817	0.11 (−0.87 to 1.10)
Mid-treatment	12.10	11.25	10.62	11.28	−1.48	.110	−0.86 (−1.86 to 0.22)
Post-treatment	11.75	11.26	10.90	12.28	−0.85	.379	−0.44 (−1.44 to 0.56)
3-month follow-up	11.59	11.61	9.28	12.26	−2.31	.046	−1.05 (−2.12 to −0.02)

LS: Least squares, CAPS: Clinician Administered PTSD Scale; PCL: PTSD Checklist for DSM-5; BDI = Beck Depression Inventory; ITQ: International Trauma Questionnaire; WHOQOL: World Health Organization Quality of Life Scale.

#### 2.3.2 Secondary outcomes (Table 3, Supplementary Table F)

For depression scores (*g* = 1.11), emotion dysregulation (*g* = 1.24), difficulties in relationships (*g* = 1.12) and environmental quality of life (*g* = −1.05), there were significant time × condition × insecurity group interactions at follow-up, indicating that participants with high insecurity who were in the STAIR-R + NET condition showed greater improvements from pre-treatment to follow-up than those in the SPS + NET condition. For emotion dysregulation, there was also a significant time × condition × insecurity group interaction at post-treatment, indicating that participants with high insecurity who were in the STAIR-R + NET condition showed greater decreases in emotion dysregulation from pre-treatment to post-treatment than those in the SPS + NET condition (*g* = 1.57).

#### 2.3.3 Protocol deviations and adverse events

There were no adverse events (defined as significant elevation in risk of harm to self or others) or serious adverse events (defined as hospitalization due to exacerbation of PTSD symptoms or elevation in suicide risk) in this study. Recruitment during this study was disrupted due to COVID-19. This, combined with funding limitations, led to a smaller sample size than projected.

## 3. Discussion

To our knowledge, this was the first study to evaluate the efficacy of emotion regulation skills training combined with trauma-focused therapy in targeting psychological symptoms amongst refugees. Our findings revealed that participants in both treatment conditions appeared to benefit equally in terms of PTSD symptom reduction. There are several potential explanations for these findings. The first is that there was inadequate statistical power in this study to detect between-group differences. Due to challenges with recruitment associated with the COVID-19 pandemic, we were unable to attain our projected sample size. Future studies investigating the relative efficacy of these interventions with a larger sample size may find greater symptom reduction is associated with one treatment modality. A second explanation for this finding is that both interventions are equally efficacious. In this study, both groups received NET, which is considered one of the leading evidence-based interventions for treating PTSD in refugees (Kip et al., 2020; Lely et al., 2019; Nosè et al., 2017; Siehl et al., 2021). Our findings add to the body of literature showing that NET is associated with reductions in PTSD symptoms when applied with refugees (although the absence of a non-NET control group precludes conclusions about the efficacy of NET compared to other interventions on the basis of these results). While participants in both treatment conditions reported significant improvements in PTSD symptoms, we did not see concomitant reductions in other secondary outcomes in this sample at follow-up. This is consistent with research evidence suggesting that NET shows the strongest effect on PTSD symptoms, with other secondary outcomes (such as depression) showing more modest gains in randomized controlled trials (Lely et al., 2019; Raghuraman et al., 2021; Siehl et al., 2021). It is also important to note that, in this study, participants received a relatively small dose of NET, with the intervention being delivered in only seven sessions, whereas several other studies have implemented NET in 10 or more sessions (Brady et al., 2021; Halvorsen & Stenmark, 2010). It may be the case that the dosage of the intervention was inadequate to promote symptom reduction beyond the primary treatment target. Further, it is notable that, while the dosage of NET was relatively modest in this study, it still exceeded the number of sessions administered in the STAIR-R phase of the intervention, which may have contributed to the lack of between-group differences between conditions, in which both received a higher number of NET sessions than STAIR-R/SPS sessions. Future research with a larger sample size, non-NET control group and that varies the number of sessions delivered would elucidate these factors. In addition, it may be useful to investigate the extent to which STAIR-R alone (i.e. not delivered in conjunction with exposure therapy) leads to symptom reduction. Recent studies have found that STAIR alone results in improved PTSD symptoms, depression symptoms, as well as emotion regulation (Cloitre et al., 2024; Ong et al., 2024), compared to control conditions. Given the large body of research attesting to the role of emotion dysregulation as a transdiagnostic mechanistic process in psychopathology (Sheppes et al., 2015), there is value in examining the extent to which STAIR-R may lead to improved outcomes for refugees as a standalone intervention.

An alternative explanation for why we did not observe overall group differences between the treatment conditions in this study may be that subgroups of participants responded differently to the two types of interventions. While the small sample size in our exploratory moderator analyses warrants tentative conclusions, we found that refugees with high insecurity appeared to benefit more from STAIR-R + NET than SPS + NET on a variety of outcomes. Specifically, compared to those who received SPS + NET, refugees with high insecurity who received STAIR-R + NET showed significantly greater improvements in self-reported (but not clinician-administered) PTSD symptoms, depression symptoms, emotion dysregulation, relationship difficulties, and environmental quality of life at the 3-month follow-up assessment. While these findings need to be replicated with a larger sample, they provide preliminary evidence that the efficacy of psychological interventions is influenced by the environmental context of refugees, and highlight the potential benefits of tailored intervention approaches (Haagen et al., 2017; Tay & Carlsson, 2022). In this study, refugees in the high insecurity group held temporary visas and/or were separated from all immediate family members. Visa insecurity and family separation have been linked to a myriad of environmental stressors, as well as heightened risk of potential catastrophic future events, including fears of being sent back to the context of persecution or the injury or death of family members living in dangerous settings (Fogden et al., 2020; Liddell et al., 2022; Nickerson et al., 2010, 2019). Accordingly, for refugees with high insecurity, treatment for PTSD takes place alongside significant ongoing threat and stressors which likely give rise to feelings of fear, anger and sadness. It may be the case that the STAIR-R intervention was more effective for refugees with high insecurity as it provided them with skills to manage the intense negative emotions arising from present stressors and future threats (Nickerson et al., 2015; Specker et al., 2024). This is supported by the finding that emotion dysregulation improved to a greater extent amongst refugees with high insecurity in STAIR-R + NET than in SPS + NET. We also found that participants with high insecurity showed greater increases in environmental quality of life following STAIR-R + NET compared to SPS + NET. These items indexed the extent to which the individual was satisfied with their environment (e.g. felt safe, had enough money, was happy with their living conditions). These findings further suggest that emotion regulation skills training may have enhanced the capacity of participants to manage these daily stressors, thus leading to improved quality of life, although this hypothesis warrants empirical evaluation.

Findings from these exploratory analyses prompt some theoretical considerations. In the field of coping research, the goodness-of-fit hypothesis suggests that emotion-focused coping strategies may be more beneficial than problem-focused coping in conditions of uncontrollable stress (Folkman & Moskowitz, 2004). This is based on the premise that problem-solving is unlikely to be effective in situations where the individual has limited influence over their external environment. In these situations, it is proposed that greater benefit may be derived from the effective management of negative emotions arising from uncontrollable stress (such as that experienced by refugees with insecure visas or separated from their family) than attempting to solve unsolvable problems. While empirical support for the goodness-of-fit hypothesis has been mixed (Boyes & French, 2012; Endler et al., 2000; Finkelstein-Fox & Park, 2019; Zakowski et al., 2001), our findings provide preliminary evidence that refugees with high insecurity may more from structured emotion regulation skills training (STAIR-R + NET), than from client-led supportive interventions with a problem-solving focus (SPS + NET). In contrast, we found that there were no group differences in treatment response for refugees with low insecurity, with participants showing global improvements in PTSD symptoms, regardless of treatment condition. It may be the case that, for refugees living with (relatively) more controllable stressors, both emotion regulation skills training and client-led supportive problem solving were effective when delivered in conjunction with NET. This highlights the interaction between environment and coping strategies in influencing psychological wellbeing (Kashyap et al., 2021).

This study had several limitations. As noted previously, this study was underpowered to detect between-group differences. Both conditions received NET, which has demonstrated efficacy for PTSD in refugees (Kip et al., 2020; Nosè et al., 2017); accordingly, it would be expected that differences between the two conditions would be relatively small. A larger sample size may have yielded a significant between-group difference in treatment outcomes for the overall sample and/or for those with low visa insecurity. Nevertheless, despite the small sample size, the differences in treatment response across conditions for those with high insecurity were consistent across self-report measures. This, combined with the magnitude of the effect sizes, provides evidence for a robust effect for those with high insecurity.

Second, we saw a discrepancy in this study between findings relating to visa insecurity and treatment condition across measure types. On all self-report measures, participants with high insecurity showed greater improvements in the STAIR-R + NET condition than the SPS + NET condition. In contrast, outcomes did not differ across conditions according to security in the clinician-administered measure (CAPS). It may be the case that the sample size in this study was not adequate to discern between-group differences on clinician-administered measures, while the self-report measures were more sensitive to differential change in this study. Further research is required to elucidate this possibility. A third limitation in the current study lies in the nature of the SPS + NET condition. This condition was selected as it closely represented treatment-as-usual for refugees receiving clinical services in Australia, however, it was a predominantly client-led problem-focused counselling as opposed to implementing a structured problem-solving approach across multiple sessions. While this enhances the ecological validity of the intervention, it limits the extent to which we can draw conclusions regarding the efficacy of problem-solving interventions.

It is important to note that this study was conducted during the COVID-19 pandemic. Research documenting changes in PTSD symptoms during the pandemic has yielded mixed results (Blendermann et al., 2024), with some studies showing PTSD symptoms increasing during this period (e.g. Daly et al., 2021), while others demonstrating decreased symptoms among clinical samples (e.g. Scarfe et al., 2023). Given the study design, it is not possible to disentangle the impact of the pandemic on PTSD symptoms with the effects of the interventions tested in this study, meaning replication of these findings to determine their robustness is necessary.

The study findings have potential research and clinical implications. Contemporary models of refugee and post-conflict mental health have highlighted the critical role of context in influencing psychological outcomes (Kashyap et al., 2021; Miller & Rasmussen, 2017; Silove, 2013). This is consistent with the recognition – in the broader literature – that different external environments, stressors and even specific situations give rise to different mechanistic pathways to mental health, thus calling for the implementation of specific strategies to promote well-being (Bonanno et al., 2024; Craske et al., 2023). This accords with a precision or personalized medicine approach to understanding and treating mental health symptoms, which highlights the importance of matching intervention to context (Ozomaro et al., 2013). Our knowledge of which treatment approaches are optimal for whom and under what circumstances is in its infancy, particularly in the field of refugee mental health. Further research investigating predictors and moderators of treatment response would advance this important line of enquiry. Although in need of replication, the finding from this study that refugees with high insecurity benefited most from emotion regulation skills training prior to trauma-focused therapy has important potential clinical implications.

This study represented the first randomized controlled trial evaluating the relative efficacy of emotion regulation skills training vs client-led supportive problem solving prior to trauma-focused therapy in refugees. Findings add to the body of evidence demonstrating that NET is associated with reductions in PTSD symptoms amongst refugees who have been exposed to war and persecution. These findings also provide preliminary evidence supporting the movement away from a one-size fits all approach to the psychological treatment of PTSD in refugees and highlighting the importance of further research into identifying the optimal intervention for refugees living in different environmental circumstances to facilitate better treatment response.

## Supplementary Material

Supplementary Tables STAIR RCT.docx

## Data Availability

Data can be obtained from the first author by reasonable request.

## Note

1 We elected to stratify due to visa insecurity as this was a key source of uncertainty for refugees. Following completion of the study, we decided to also include participants separated from all immediate family in the high insecurity group due to similar experiences of ongoing uncertainty across the two groups and to increase the sample size in moderation analyses.
