# Supplementary material for: Emotion regulation skills training as an adjunctive treatment to narrative exposure therapy for posttraumatic stress disorder (PTSD) in refugees: a pilot randomized controlled trial
Source: Eur J Psychotraumatol. 2026 Apr 8;17(1):2648941. doi: 10.1080/20008066.2026.2648941 (PMC13063331; doi:10.1080/20008066.2026.2648941)
Supplement: Supplementary Tables STAIR RCT.docx [file ZEPT_A_2648941_SM7601.docx]

Supplementary Figure A. STAIR Consort Diagram

Screens completed (N=394)

Excluded (n=37)

♦  Not meeting inclusion criteria (n=20)

♦  Declined to participate (n=17)

Randomized (n=71)

Baseline completed (N=108)

## Enrollment

3-month follow up clinical interview (n=29)

- Withdrawn/Lost (n=6; 17.1%)
- Total attrition (n=10; 28.6%)

3-month follow up online measures (n=24)

- Withdrawn/Lost (n=11; 31.4%)
- Total attrition (n=18; 51.4%)

3-month follow up clinical interview (n=30)

- Withdrawn/Lost (n=6; 16.7%)
- Total attrition (n=9; 25%)

3-month follow up online measures (n=26)

- Withdrawn/Lost (n=10; 27.8%)
- Total attrition (n=13; 36.1%)

## Follow Up

Mid-treatment online measures (n=31)

- Withdrawn/Lost (n=1; 2.9%)
- Missed (n=3)

Mid-treatment online measures (n=28)

- Withdrawn/Lost (n=1; 2.8%)
- Missed (n=7)

Allocated to STAIR + NET (n=35)

Allocated to SPS + NET (n=36)

## Allocation

Post-treatment clinical interview (n=30)

- Withdrawn/Lost (n=3; 8.6%)
- Total attrition (n=4; 11.4%)
- Missed (n=2)

Post-treatment online measures (n=30)

- Withdrawn/Lost (n=3; 8.6%)
- Total attrition (n=4; 11.4%)
- Missed (n=2)

Post-treatment clinical interview (n=30)

- Withdrawn/Lost (n=2; 5.6%)
- Total attrition (n=3; 8.3%)
- Missed (n=4)

Post-treatment online measures (n=29)

- Withdrawn/Lost (n=2; 5.6%)
- Total attrition (n=3; 8.3%)
- Missed (n=5)

Supplementary Table A. Comparison of participants who completed 3-month follow-up assessment and those who did not complete 3-month follow-up assessment.

|  | Completed  3-month follow-up  N = 59 | Did not complete  3-month follow-up  N = 12 | *t* or *χ^2^* |
| --- | --- | --- | --- |
| Age, years | 46.81 (10.98) | 46.67 (13.18) | *t*(69) = -0.04, *p* = .485 |
| Sex |  |  |  |
| Female | 41 (87.2%) | 6 (12.8%) | *χ^2^*(1) = 1.69, *p* = .315 |
| Male | 18 (75.0%) | 6 (25.0%) |  |
| PTE Exposure | 11.37 (4.21) | 14.00 (4.54) | *t*(63) = 1.86, *p* = .067 |
| Time in Australia | 4.34 (5.01) | 3.87 (3.31) | *t*(69) = -0.31, *p* = .377 |
| Insecurity status |  |  |  |
| High insecurity status | 10 (62.5%) | 6 (37.5%) | *χ^2^*(1) = 5.49, *p* = .019 |
| Low insecurity status | 45 (88.2%) | 6 (11.8%) |  |
| Language |  |  |  |
| Arabic | 54 (81.8%) | 12 (18.2%) | *χ^2^*(2) = 1.09, *p* = .579 |
| Farsi | 2 (100.0%) | 0 (0%) |  |
| English | 1 (100.0%) | 0 (0%) |  |
| Medication |  |  |  |
| Yes | 18 (78.3%) | 5 (21.7%) | *χ^2^*(1) = 2.11, *p* = .145 |
| No | 25 (92.6%) | 2 (7.4%) |  |
| Therapy with interpreter |  |  |  |
| Yes | 58 (84.1%) | 11 (15.9%) | *χ^2^*(1) = 1.61, *p* = .205 |
| No | 1 (50.0%) | 1 (50.0%) |  |
| Therapy modality |  |  |  |
| Face-to-face only | 11 (78.6%) | 3 (21.4%) | *χ^2^*(1) = 0.25, *p* = .693 |
| Online or hybrid | 48 (84.2%) | 9 (15.8%) |  |
| PTSD symptoms  (clinician-administered) | 38.86 (9.03) | 42.08 (10.33) | *t*(69) = 1.10, *p* = .276 |
| PTSD symptoms  (self-report) | 49.85 (16.71) | 52.91 (15.55) | *t*(64) = 0.56, *p* = .578 |
| Depression symptoms | 29.82 (12.72) | 32.27 (13.18) | *t*(64) = 0.58, *p* = .563 |
| Emotion dysregulation | 4.88 (1.57) | 4.90 (2.77) | *t*(64) = 0.03, *p* = .980 |
| Relationship difficulties | 72.96 (14.72) | 80.45 (13.34) | *t*(56) = 1.76, *p* = .083 |
| Environmental quality of life | 10.78 (2.71) | 10.95 (2.14) | *t*(63) = 0.20, *p* = .839 |

Supplementary Table B. Mean, standard deviation and range of outcome variables, and within-group effect sizes according treatment condition.

|  | Overall sample  (n = 71) | | STAIR-R+NET  (n = 35) | | | SPS+NET  (n = 36) | | |
| --- | --- | --- | --- | --- | --- | --- | --- | --- |
|  | Mean (SD) | Range | Mean (SD) | Range | Hedges g (from baseline) | Mean (SD) | Range | Hedges g (from baseline) |
| CAPS score |  |  |  |  |  |  |  |  |
| Baseline | 39.46 (9.33) | 22 to 64 | 38.80 (8.92) | 22 to 62 |  | 40.11 (9.79) | 22 to 64 |  |
| Post-treatment | 23.58 (12.20) | 1 to 50 | 22.93 (12.42) | 1 to 50 | -1.61 | 24.23 (12.15) | 4 to 48 | -1.64 |
| 3-month follow-up | 26.66 (13.58) | 0 to 51 | 27.41 (14.06) | 3 to 51 | -1.14 | 25.93 (13.30) | 0 to 51 | -1.54 |
|  |  |  |  |  |  |  |  |  |
| PCL score |  |  |  |  |  |  |  |  |
| Baseline | 50.36 (16.45) | 3 to 79 | 3 to 79 | 3 to 79 |  | 52.00 (13.82) | 20 to 78 |  |
| Mid-treatment | 44.22 (18.77) | 0 to 74 | 0 to 74 | 0 to 74 | -0.25 | 43.82 (16.47) | 7 to 67 | -0.42 |
| Post-treatment | 40.31 (18.49) | 1 to 77 | 1 to 77 | 1 to 77 | -0.45 | 40.56 (15.94) | 4 to 66 | -0.68 |
| 3-month follow-up | 42.78 (18.28) | 5 to 73 | 5 to 73 | 5 to 73 | -0.49 | 45.84 (15.90) | 7 to 73 | -0.44 |
|  |  |  |  |  |  |  |  |  |
| BDI score |  |  |  |  |  |  |  |  |
| Baseline | 30.23 (12.73) | 5 to 56 | 31.50 (14.06) | 5 to 56 |  | 29.03 (11.42) | 8 to 52 |  |
| Mid-treatment | 28.36 (15.06) | 0 to 56 | 29.93 (16.08) | 0 to 56 | -0.16 | 26.54 (13.88) | 3 to 55 | -0.33 |
| Post-treatment | 27.38 (13.63) | 2 to 59 | 28.76 (15.00) | 2 to 59 | -0.30 | 26.11 (12.38) | 7 to 50 | -0.27 |
| 3-month follow-up | 27.57 (15.29) | 1 to 60 | 26.46 (15.91) | 2 to 55 | -0.35 | 28.64 (14.91) | 1 to 60 | -0.16 |
|  |  |  |  |  |  |  |  |  |
| ITQ- Affective Dysregulation Score | | | | | | | | |
| Baseline | 4.89 (2.05) | 1 to 8 | 5.24 (2.23) | 1 to 8 |  | 4.56 (1.85) | 1 8 |  |
| Mid-treatment | 4.27 (2.29) | 0 to 8 | 4.62 (2.55) | 0 to 8 | -0.28 | 3.96 (2.05) | 0 to 8 | -0.32 |
| Post-treatment | 4.02 (2.11) | 0 to 8 | 4.04 (2.51) | 0 to 8 | -0.61 | 4.00 (1.72) | 0 to 7 | -0.35 |
| 3-month follow-up | 4.04 (2.30) | 0 to 8 | 4.00 (2.93) | 0 to 8 | -0.61 | 4.08 (1.61) | 1 to 6 | -0.29 |
| ITQ-Difficulties in Relationships Score | | | | | | | | |
| Baseline | 4.57 (2.25) | 0 to 8 | 4.90 (2.44) | 0 to 8 |  | 4.28 (2.05) | 0 to 8 |  |
| Mid-treatment | 4.02 (2.35) | 0 to 8 | 4.12 (2.31) | 0 to 8 | -0.31 | 3.93 (2.42) | 0 to 8 | -0.18 |
| Post-treatment | 3.69 (2.23) | 0 to 8 | 3.70 (2.42) | 0 to 8 | -0.52 | 3.69 (2.09) | 0 to 8 | -0.25 |
| 3-month follow-up | 4.09 (2.57) | 0 to 8 | 3.95 (2.82) | 0 to 8 | -0.37 | 4.21 (2.38) | 0 to 8 | -0.06 |
| WHOQOL Environmental Domain score | | | | | | | | |
| Baseline | 10.81 (2.61) | 5 to 18 | 10.87 (2.82) | 6 to 17.5 |  | 10.75 (2.44) | 5 to 18 |  |
| Mid-treatment | 11.27 (2.57) | 6 to 19 | 11.47 (3.02) | 6 to 19 | 0.26 | 11.06 (2.00) | 8 to 17 | 0.12 |
| Post-treatment | 11.43 (2.35) | 6 to 19 | 11.25 (2.81) | 6 to 19 | 0.20 | 11.61 (1.80) | 8.5 to 15 | 0.40 |
| 3-month follow-up | 11.35 (2.94) | 4.5 to 19 | 11.54 (3.11) | 6.5 to 19 | 0.26 | 11.17 (2.80) | 4.50 to 16 | 0.28 |

CAPS = Clinician Administered PTSD Scale, PCL = PTSD Checklist for DSM-5, BDI= Beck Depression Inventory, ITQ = International Trauma Questionnaire, WHOQOL = World Health Organization Quality of Life Scale.

Supplementary Table C. Results of mixed models analysis investigating differential change in symptoms over time according to treatment condition.

|  | Estimate | SE | df | *t* | *p*-value | 95% CI Lo | 95% CI Hi |
| --- | --- | --- | --- | --- | --- | --- | --- |
| CAPS score |  |  |  |  |  |  |  |
| Intercept | 40.11 | 1.96 | 130.35 | 20.48 | < .001 | 36.3 | 43.92 |
| Time |  |  |  |  |  |  |  |
| Pre-treatment | - | - | - | - | - | - | - |
| Post-treatment | -15.50 | 2.13 | 121.22 | -7.29 | < .001 | -19.64 | -11.37 |
| 3-month follow-up | -14.50 | 2.13 | 121.22 | -6.82 | < .001 | -18.63 | -10.37 |
| Condition |  |  |  |  |  |  |  |
| SPS+NET | - | - | - | - | - | - | - |
| STAIR+NET | -1.31 | 2.79 | 130.35 | -0.47 | .639 | -6.73 | 4.11 |
| Time x condition |  |  |  |  |  |  |  |
| Post-treatment SPS+NET | - | - | - | - | - | - | - |
| Post-treatment STAIR+NET | 0.34 | 3.02 | 121.36 | 0.11 | .909 | -5.52 | 6.20 |
| 3-month follow-up -SPS+NET | - | - | - | - | - | - | - |
| 3-month follow-up - STAIR+NET | 3.75 | 3.04 | 121.63 | 1.24 | .219 | -2.16 | 9.64 |
|  |  |  |  |  |  |  |  |
| PCL score |  |  |  |  |  |  |  |
| Intercept | 52.02 | 3.00 | 106.63 | 17.37 | < .001 | 46.09 | 57.96 |
| Time |  |  |  |  |  |  |  |
| Pre-treatment | - | - | - | - | - | - | - |
| Mid-treatment | -7.04 | 2.52 | 159.40 | -2.79 | .006 | -12.02 | -2.06 |
| Post-treatment | -11.25 | 2.57 | 160.54 | -4.37 | <.001 | -16.33 | -6.17 |
| 3-month follow-up | -7.36 | 2.64 | 160.93 | -2.78 | .006 | -12.47 | -2.14 |
| Condition |  |  |  |  |  |  |  |
| SPS+NET | - | - | - | - | - | - | - |
| STAIR+NET | -3.17 | 4.30 | 106.82 | -0.74 | .463 | -11.70 | 5.36 |
| Time x condition |  |  |  |  |  |  |  |
| Mid-treatment SPS+NET | - | - | - | - | - | - | - |
| Mid-treatment STAIR+NET | 2.94 | 3.51 | 157.55 | 0.84 | .403 | -3.99 | 9.86 |
| Post-treatment SPS+NET | - | - | - | - | - | - | - |
| Post-treatment STAIR+NET | 3.66 | 3.61 | 158.72 | 1.05 | .297 | -3.35 | 10.90 |
| 3-month follow-up - SPS+NET | - | - | - | - | - | - | - |
| 3-month follow-up - STAIR+NET | -0.80 | 3.78 | 160.50 | -0.21 | .832 | -8.26 | 6.66 |
|  |  |  |  |  |  |  |  |
| BDI score |  |  |  |  |  |  |  |
| Intercept | 29.19 | 2.39 | 12.21 | 9.07 | <.001 | 24.54 | 33.85 |
| Time |  |  |  |  |  |  |  |
| Pre-treatment | - | - | - | - | - | - | - |
| Mid-treatment | -4.21 | 0.22 | 153.39 | -1.94 | .054 | -8.49 | 0.07 |
| Post-treatment | -3.51 | 2.15 | 154.08 | -1.63 | .105 | -7.76 | 0.74 |
| 3-month follow-up | -2.10 | 2.21 | 154.69 | -0.95 | .344 | -6.47 | 2.27 |
| Condition |  |  |  |  |  |  |  |
| SPS+NET | - | - | - | - | - | - | - |
| STAIR+NET | 2.57 | 3.44 | 109.41 | 0.75 | .456 | -4.24 | 9.38 |
| Time x condition |  |  |  |  |  |  |  |
| Mid-treatment SPS+NET | - | - | - | - | - | - | - |
| Mid-treatment STAIR+NET | 2.09 | 2.99 | 151.64 | 0.70 | .485 | -3.82 | 8.01 |
| Post-treatment SPS+NET | - | - | - | - | - | - | - |
| Post-treatment STAIR+NET | -0.31 | 3.08 | 152.89 | -0.10 | .920 | -6.40 | 5.78 |
| 3-month follow-up -SPS+NET | - | - | - | - | - | - | - |
| 3-month follow-up - STAIR+NET | -2.40 | 3.16 | 154.53 | -0.76 | .449 | -9.65 | 3.85 |
|  |  |  |  |  |  |  |  |
| ITQ - Emotion dysregulation | |  |  |  |  |  |  |
| Intercept | 4.57 | 0.38 | 110.09 | 11.91 | <. 001 | 3.81 | 5.33 |
| Time |  |  |  |  |  |  |  |
| Pre-treatment | - | - | - | - | - | - | - |
| Mid-treatment | -0.67 | 0.37 | 140.93 | -1.82 | .070 | -1.37 | 0.06 |
| Post-treatment | -0.72 | 0.38 | 142.98 | -1.90 | .059 | -1.46 | 0.03 |
| 3-month follow-up | -0.61 | 0.39 | 143.54 | -1.57 | .118 | -1.38 | 0.16 |
| Condition |  |  |  |  |  |  |  |
| SPS+NET | - | - | - | - | - | - | - |
| STAIR+NET | 0.70 | 0.56 | 110.33 | 1.26 | .211 | -0.40 | 1.80 |
| Time x condition |  |  |  |  |  |  |  |
| Mid-treatment SPS+NET | - | - | - | - | - | - | - |
| Mid-treatment STAIR+NET | 0.08 | 0.54 | 142.07 | 0.14 | .887 | -0.98 | 1.14 |
| Post-treatment SPS+NET | - | - | - | - | - | - | - |
| Post-treatment STAIR+NET | -0.53 | 0.55 | 143.31 | -0.98 | .328 | -1.62 | 0.55 |
| 3-month follow-up -SPS+NET | - | - | - | - | - | - | - |
| 3-month follow-up - STAIR+NET | -0.65 | 0.57 | 145.54 | -1.13 | .259 | -1.769 | 0.48 |
|  |  |  |  |  |  |  |  |
| ITQ - Relationship difficulties | |  |  |  |  |  |  |
| Intercept | 4.26 | 0.41 | 101..48 | 10.30 | < .001 | 3.44 | 5.08 |
| Time |  |  |  |  |  |  |  |
| Pre-treatment | - | - | - | - | - | - | - |
| Mid-treatment | -0.42 | 0.37 | 139.67 | -1.13 | .262 | -1.14 | 0.31 |
| Post-treatment | -0.57 | 0.38 | 141.42 | -1.49 | .140 | -1.32 | 0.19 |
| 3-month follow-up | -0.14 | 0.39 | 141.88 | -0.36 | .717 | -0.92 | 0.63 |
| Condition |  |  |  |  |  |  |  |
| SPS+NET | - | - | - | - | - | - | - |
| STAIR+NET | 0.77 | 0.60 | 101.75 | 1.28 | .204 | -0.42 | 1.96 |
| Time x condition |  |  |  |  |  |  |  |
| Mid-treatment SPS+NET | - | - | - | - | - | - | - |
| Mid-treatment STAIR+NET | -0.29 | 0.54 | 140.70 | -0.54 | .590 | -1.36 | 0.78 |
| Post-treatment SPS+NET | - | - | - | - | - | - | - |
| Post-treatment STAIR+NET | -0.62 | 0.55 | 141.76 | -1.12 | .260 | -1.72 | 0.48 |
| 3-month follow-up -SPS+NET | - | - | - | - | - | - | - |
| 3-month follow-up - STAIR+NET | -0.69 | 0.58 | 143.74 | -1.20 | .231 | -1.83 | 0.45 |
|  |  |  |  |  |  |  |  |
| WHO – Environmental QOL | |  |  |  |  |  |  |
| Intercept | 10.64 | 0.45 | 101.53 | 23.48 | < .001 | 9.74 | 11.54 |
| Time |  |  |  |  |  |  |  |
| Pre-treatment | - | - | - | - | - | - | - |
| Mid-treatment | 0.34 | 0.39 | 151.90 | 0.87 | .385 | -0.43 | 1.10 |
| Post-treatment | 1.06 | 0.39 | 152.71 | 2.73 | .007 | 0.29 | 1.83 |
| 3-month follow-up | 0.75 | 0.41 | 153.47 | 1.85 | .067 | -0.05 | 1.55 |
| Condition |  |  |  |  |  |  |  |
| SPS+NET | - | - | - | - | - | - | - |
| STAIR+NET | 0.22 | 0.65 | 102.84 | 0.34 | .736 | -1.07 | 1.52 |
| Time x condition |  |  |  |  |  |  |  |
| Mid-treatment SPS+NET | - | - | - | - | - | - | - |
| Mid-treatment STAIR+NET | 0.36 | 0.54 | 150.06 | 0.66 | .511 | -0.71 | 1.42 |
| Post-treatment SPS+NET | - | - | - | - | - | - | - |
| Post-treatment STAIR+NET | -0.54 | 0.55 | 151.02 | -0.98 | .328 | -1.62 | 0.55 |
| 3-month follow-up SPS+NET | - | - | - | - | - | - | - |
| 3-month follow-up - STAIR+NET | -0.08 | 0.58 | 153.07 | -0.14 | .893 | -1.22 | 1.06 |

CAPS = Clinician Administered PTSD Scale, PCL = PTSD Checklist for DSM-5, BDI= Beck Depression Inventory, ITQ = International Trauma Questionnaire, WHOQOL = World Health Organization Quality of Life Scale, CI = Confidence Interval.

Supplementary Table D. Demographic characteristics of participants with high insecurity status and low insecurity status.

|  | Overall sample  N = 71 | High  insecurity  status  n = 16 | Low  insecurity status  n = 55 | *t* or *χ^2^* |
| --- | --- | --- | --- | --- |
| Age, years | 46.79 (11.28) | 45.33 (10.31) | 47.21 (11.60) | *t*(69) = -0.58, *p* = .561 |
| Sex |  |  |  |  |
| Female | 47 (66.2%) | 9 (56.3%) | 38 (69.1%) | *χ^2^*(1) = 0.91, *p* = .339 |
| Male | 24 (33.8%) | 7 (43.8%) | 17 (30.9%) |  |
| PTE Exposure | 11.82 (4.35) | 13.79 (5.48) | 11.27 (3.88) | *t*(63) = 1.95, *p* = .028 |
| Time in Australia | 4.26 (4.75) | 7.14 (6.15) | 3.42 (3.95) | *t*(69) = 2.90, *p* = .003 |
| Language |  |  |  |  |
| Arabic | 66 (93.0%) | 14 (87.6%) | 52 (94.5%) | *χ^2^*(2) = 1.13, *p* = .568 |
| Farsi | 2 (2.8%) | 1 (6.3%) | 2 (3.6%) |  |
| English | 3 (4.2%) | 1 (6.3%) | 2 (1.8%) |  |
| Medication |  |  |  |  |
| Yes | 23 (46.0%) | 7 (36.4%) | 16 (59.0%) | *χ^2^*(1) = 0.65, *p* = .419 |
| No | 27 (54.0%) | 4 (63.6%) | 23 (41.0%) |  |
| Therapy with interpreter |  |  |  |  |
| Yes | 69 (97.2%) | 15 (93.8%) | 54 (98.2%) | *χ^2^*(1) = 0.89, *p* = .346 |
| No | 2 (2.8%) | 1 (6.3%) | 1 (1.8%) |  |
| Therapy modality |  |  |  |  |
| Face-to-face only | 14 (19.7%) | 1 (6.3%) | 12 (21.8%) | *χ^2^*(1) = 2.01*,* *p* = .156 |
| Online or hybrid | 57 (80.3%) | 15 (93.8%) | 43 (78.2%) |  |
| Mean number of sessions attended | 11.27 (3.47) | 10.81 (3.52) | 11.40 (3.48) | *t*(69) = -0.59, *p* = .278 |
| PTSD symptoms  (clinician-administered) | 39.41 (9.27) | 44.32 (8.56) | 38.05 (9.13) | *t*(69) = 2.45, *p* = .009 |
| PTSD symptoms  (self-report) | 50.36 (12.73) | 58.40 (14.05) | 48.00 (16.48) | *t*(64) = 2.22, *p* = .015 |
| Depression symptoms | 30.23 (12.73) | 38.20 (12.16) | 27.88 (12.02) | *t*(64) = 2.92, *p* = .002 |
| Emotion dysregulation | 4.89 (2.05) | 4.85 (2.30) | 4.50 (2.25) | *t*(59) = 0.68, *p* = .249 |
| Relationship difficulties | 4.57 (2.25) | 4.90 (2.44) | 4.28 (2.05) | *t*(59) = 0.49, *p* = .313 |
| Environmental quality of life | 10.81 (2.61) | 10.33 (2.01) | 10.95 (2.76) | *t*(63) = -0.80, *p* = .175 |

SD = standard deviation; PTE = potentially traumatic events; PTSD = posttraumatic stress disorder

Supplementary Table E. Mean, standard deviation and range of outcome variables according to insecurity status and treatment condition.

|  | | | | STAIR+  NET High insecurity  (n = 7) | | | | STAIR+  NET Low  Insecurity (n = 26) | | | | | | SPS+NET  High insecurity (n = 9) | | | | SPS+NET  Low insecurity (n = 25) | | | |
| --- | --- | --- | --- | --- | --- | --- | --- | --- | --- | --- | --- | --- | --- | --- | --- | --- | --- | --- | --- | --- | --- |
|  | Mean (SD) | | | | Range | | Mean (SD) | | | Range | | Mean (SD) | | | Range | | Mean (SD) | | | | Range |
| CAPS score | |  | | | |  | | |  | | | |  | | |  | | | |  | |
| Baseline | 40.29 (7.89) | | | | 30.00 to 51.00 | | 38.43 (9.26) | | | 22.00 to 62.00 | | 47.44 (8.09) | | | 35.00 to 64.00 | | 37.67 (9.16) | | | | 22.00 to 60.00 |
| Post-treatment | 25.60 (8.76) | | | | 17.00 to 38.00 | | 22.40 (13.11) | | | 1.00 to 50.00 | | 25.71 (13.38) | | | 5.00 to 40.00 | | 23.78 (12.04) | | | | 4.00 to 48.00 |
| 3-month follow-up | 29.00 (8.29) | | | | 22.00 to 41.00 | | 27.16 (14.89) | | | 3.00 to 51.00 | | 34.33 (9.44) | | | 24.00 to 48.00 | | 23.83 (13.44) | | | | 0.00 to 51.00 |
|  |  | | | |  | |  | | |  | |  | | |  | |  | | | |  |
| PCL score | |  | | | |  | | |  | | | |  | | |  | | | |  | |
| Baseline | 60.83 (19.53) | | | | 36.00 to 79.00 | | 45.81 (17.99) | | | 3.00 to 77.00 | | 56.78 (9.98) | | | 46.00 to 78.00 | | 50.28 (14.75) | | | | 20.00 to 77.00 |
| Mid-treatment | 48.17 (18.66) | | | | 28.00 to 72.00 | | 43.72 (21.66) | | | 0.00 to 74.00 | | 49.75 (16.35) | | | 13.00 to 66.00 | | 41.45 (16.32) | | | | 7.00 to 67.00 |
| Post-treatment | 39.20 (17.25) | | | | 18.00 to 54.00 | | 40.26 (22.02) | | | 1.00 to 77.00 | | 51.71 (7.39) | | | 43.00 to 66.00 | | 36.65 (16.39) | | | | 4.00 to 66.00 |
| 3-month follow-up | 40.75 (9.74) | | | | 27.00 to 49.00 | | 39.35 (22.00) | | | 5.00 to 73.00 | | 59.17 (11.55) | | | 44.00 to 73.00 | | 41.63 (14.90) | | | | 7.00 to 58.00 |
|  |  | | | |  | |  | | |  | |  | | |  | |  | | | |  |
| BDI score | |  | | | |  | | |  | | | |  | | |  | | | |  | |
| Baseline | 41.17 (13.98) | | | | 20.00 to 56.00 | | 29.27 (13.36) | | | 5.00 to 55.00 | | 36.22 (11.21) | | | 14.00 to 49.00 | | 26.44 (10.53) | | | | 8.00 to 52.00 |
| Mid-treatment | 33.33 (18.58) | | | | 11.00 to 56.00 | | 29.08 (15.72) | | | 0.00 to 54.00 | | 28.62 (19.17) | | | 3.00 to 55.00 | | 25.61 (11.36) | | | | 7.00 to 44.00 |
| Post-treatment | 33.00 (15.94) | | | | 12.00 to 48.00 | | 27.70 (15.00) | | | 2.00 to 59.00 | | 36.71 (9.96) | | | 22.00 to 47.00 | | 22.40 (11.06) | | | | 7.00 to 50.00 |
| 3-month follow-up | 34.50 (17.64) | | | | 12.00 to 55.00 | | 24.85 (15.53) | | | 2.00 to 53.00 | | 46.00 (10.99) | | | 32.00 to 60.00 | | 23.16 (11.44) | | | | 1.00 to 45.00 |
| ITQ – Emotion dysregulation score | | |  | | | |  | | | |  | | | |  | | | |  | | |
| Baseline | 6.50 (1.29) | | | | 5.00 to 8.00 | | 5.04 (2.30) | | | 1.00 to 8.00 | | 4.67 (2.00) | | | 1.00 to 8.00 | | 4.52 (1.83) | | | | 1.00 to 7.00 |
| Mid-treatment | 3.67 (3.06) | | | | 1.00 to 7.00 | | 4.76 (2.53) | | | 0.00 to 8.00 | | 4.00 (2.14) | | | 0.00 to 7.00 | | 3.95 (2.06) | | | | 0.00 to 8.00 |
| Post-treatment | 3.00 (1.41) | | | | 2.00 to 4.00 | | 4.14 (2.59) | | | 0.00 to 8.00 | | 4.86 (0.69) | | | 4.00 to 6.00 | | 3.68 (1.89) | | | | 0.00 to 7.00 |
| 3-month follow-up | 4.00 (4.24) | | | | 1.00 to 7.00 | | 4.00 (2.92) | | | 0.00 to 8.00 | | 5.17 (0.98) | | | 4.00 to 6.00 | | 3.72 (1.64) | | | | 1.00 to 6.00 |
| ITQ – Difficulties in Relationships Score | | |  | | | |  | | | |  | | | |  | | | |  | | |
| Baseline | 6.25 (2.36) | | | | 3.00 to 8.00 | | 4.68 (2.43) | | | 0.00 to 8.00 | | 4.22 (2.11) | | | 0.00 to 7.00 | | 4.30 (2.08) | | | | 0.00 to 8.00 |
| Mid-treatment | 4.67 (2.08) | | | | 3.00 to 7.00 | | 4.05 (2.38) | | | 0.00 to 8.00 | | 4.12 (2.85) | | | 0.00 to 8.00 | | 3.85 (2.30) | | | | 1.00 to 8.00 |
| Post-treatment | 4.00 (1.41) | | | | 3.00 to 5.00 | | 3.67 (2.52) | | | 0.00 to 8.00 | | 4.71 (2.14) | | | 2.00 to 8.00 | | 3.32 (2.00) | | | | 0.00 to 7.00 |
| 3-month follow-up | 4.50 (3.54) | | | | 2.00 to 7.00 | | 3.89 (2.85) | | | 0.00 to 8.00 | | 6.00 (1.90) | | | 3.00 to 8.00 | | 3.61 (2.25) | | | | 0.00 to 8.00 |
| WHOQOL- Environmental subscale | | |  | | | |  | | | |  | | | |  | | | |  | | |
| Baseline | 9.67 (2.14) | | | | 6.00 to 12.00 | | 11.16 (2.92) | | | 6.00 to 17.50 | | 10.78 (1.92) | | | 7.00 to 14.00 | | 10.74 (2.63) | | | | 5.00 to 18.00 |
| Mid-treatment | 11.17 (2.91) | | | | 7.00 to 16.00 | | 11.54 (3.10) | | | 6.00 to 19.00 | | 10.69 (2.91) | | | 8.00 to 17.00 | | 11.21 (1.54) | | | | 8.50 to 13.50 |
| Post-treatment | 10.80 (2.25) | | | | 7.00 to 12.50 | | 11.35 (2.95) | | | 6.00 to 19.00 | | 11.00 (2.43) | | | 8.50 to 15.00 | | 11.82 (1.55) | | | | 9.00 to 14.50 |
| 3-month follow-up | 10.25 (1.19) | | | | 8.50 to 11.00 | | 11.80 (3.33) | | | 6.50 to 19.00 | | 9.00 (3.77) | | | 4.50 to 14.50 | | 11.74 (2.28) | | | | 8.50 to 16.00 |

CAPS = Clinician Administered PTSD Scale, PCL = PTSD Checklist for DSM-5, BDI= Beck Depression Inventory, ITQ = International Trauma Questionnaire, WHOQOL = World Health Organization Quality of Life Scale.

Supplementary Table F. Results of mixed models analysis investigating differential change in symptoms over time according to treatment condition and insecurity status.

|  | Est | SE | df | *t* | *p*-value | 95% CI Lo | 95% CI Hi |
| --- | --- | --- | --- | --- | --- | --- | --- |
| CAPS score |  |  |  |  |  |  |  |
| Intercept | 39.07 | 1.84 | 160.87 | 21.27 | < .001 | 35.57 | 42.56 |
| Time |  |  |  |  |  |  |  |
| Pre-treatment | - | - | - | - | - | - | - |
| Post-treatment | -13.68 | 2.38 | 121.94 | -5.75 | < .001 | -18.23 | -9.15 |
| 3-month follow-up | -14.25 | 2.35 | 120.74 | -6.08 | < .001 | -18.72 | -9.76 |
| Condition |  |  |  |  |  |  |  |
| SPS+NET | - | - | - | - | - | - | - |
| STAIR+NET | 0.17 | 2.56 | 161.55 | 0.07 | .947 | -4.7 | 5.04 |
| Time x condition |  |  |  |  |  |  |  |
| Post-treatment SPS+NET | - | - | - | - | - | - | - |
| Post-treatment STAIR+NET | -1.74 | 3.31 | 121.54 | -0.53 | .600 | -8.05 | 4.59 |
| 3-month follow-up - SPS+NET | - | - | - | - | - | - | - |
| 3-month follow-up - STAIR+NET | 3.74 | 3.29 | 120.90 | 1.14 | .258 | -2.56 | 10.00 |
| Insecurity status |  |  |  |  |  |  |  |
| Low insecurity | - | - | - | - | - | - | - |
| High insecurity | 2.17 | 3.78 | 156.11 | 0.58 | .566 | -5.00 | 9.35 |
| Time x insecurity status |  |  |  |  |  |  |  |
| Post-treatment low insecurity | - | - | - | - | - | - | - |
| Post-treatment high insecurity | -8.04 | 4.87 | 126.26 | -1.65 | .101 | -17.32 | 1.27 |
| 3-month follow-up low insecurity | - | - | - | - | - | - | - |
| 3-month follow-up high insecurity | -0.13 | 5.05 | 129.01 | -0.03 | .979 | -9.81 | 9.48 |
| Condition x insecurity |  |  |  |  |  |  |  |
| SPS+NET low insecurity | - | - | - | - | - | - | - |
| SPS+NET high insecurity | - | - | - | - | - | - | - |
| STAIR+NET low insecurity | - | - | - | - | - | - | - |
| STAIR+NET high insecurity | -1.76 | 5.48 | 159.96 | -0.32 | .749 | -12.18 | 8.66 |
| Time x condition x insecurity status | | |  |  |  |  |  |
| Post-treatment SPS+NET low insecurity | - | - | - | - | - | - | - |
| Post-treatment SPS+NET high insecurity | - | - | - | - | - | - | - |
| Post-treatment STAIR+NET low insecurity | - | - | - | - | - | - | - |
| Post-treatment STAIR+NET high insecurity | 11.83 | 7.32 | 127.97 | 1.62 | .109 | -2.17 | 25.79 |
| 3-month follow-up SPS+NET low insecurity | - | - | - | - | - | - | - |
| 3-month follow-up SPS+NET high insecurity | - | - | - | - | - | - | - |
| 3-month follow-up STAIR+NET low insecurity | - | - | - | - | - | - | - |
| 3-month follow-up STAIR + NET high insecurity | 1.19 | 7.72 | 131.78 | 0.15 | .878 | -13.54 | 15.93 |
| Baseline CAPS score | 0.78 | 0.10 | 69.99 | 8.11 | < .001 | 0.59 | 0.96 |
|  |  |  |  |  |  |  |  |
| PCL score |  |  |  |  |  |  |  |
| Intercept | 33.34 | 2.74 | 123.25 | 12.17 | < .001 | 27.92 | 38.76 |
| Time |  |  |  |  |  |  |  |
| Pre-treatment | - | - | - | - | - | - | - |
| Mid-treatment | -6.66 | 2.93 | 154.38 | -2.27 | .024 | -12.45 | -0.87 |
| Post-treatment | -13.23 | 2.98 | 155.28 | -4.44 | < .001 | -19.12 | -7.34 |
| 3-month follow-up | -8.25 | 3.04 | 156.08 | -2.71 | .007 | -14.25 | 2.24 |
| Condition |  |  |  |  |  |  |  |
| SPS+NET | - | - | - | - | - | - | - |
| STAIR+NET | 0.70 | 3.31 | 153.90 | -0.21 | .834 | -7.24 | 5.85 |
| Time x condition |  |  |  |  |  |  |  |
| Mid-treatment SPS+NET | - | - | - | - | - | - | - |
| Mid-treatment STAIR+NET | 4.38 | 3.97 | 151.55 | 1.10 | .272 | -3.47 | 12.23 |
| Post-treatment SPS+NET | - | - | - | - | - | - | - |
| Post-treatment STAIR+NET | 9.09 | 4.07 | 153.06 | 2.24 | .027 | 1.06 | 17.12 |
| 3-month follow-up SPS+NET | - | - | - | - | - | - | - |
| 3-moth follow-up - STAIR+NET | 3.05 | 4.24 | 155.03 | -0.72 | .473 | -5.33 | 11.43 |
| Insecurity status |  |  |  |  |  |  |  |
| Low insecurity | - | - | - | - | - | - | - |
| High insecurity | 1.01 | 4.60 | 153.79 | 0.22 | .827 | -8.08 | 10.09 |
| Time x insecurity status |  |  |  |  |  |  |  |
| Mid-treatment low insecurity | - | - | - | - | - | - | - |
| Mid-treatment high insecurity | 0.33 | 5.48 | 152.49 | 0.06 | .952 | -10.50 | 11.16 |
| Post-treatment low insecurity | - | - | - | - | - | - | - |
| Post-treatment high insecurity | 7.10 | 5.69 | 155.00 | 1.25 | .214 | -4.14 | 18.34 |
| 3-month follow-up low insecurity | - | - | - | - | - | - | - |
| 3-month follow-up high insecurity | 8.48 | 5.94 | 156.55 | 1.43 | .155 | -3.25 | 20.22 |
| Condition x insecurity |  |  |  |  |  |  |  |
| SPS+NET low insecurity | - | - | - | - | - | - | - |
| SPS+NET high insecurity | - | - | - | - | - | - | - |
| STAIR+NET low insecurity | - | - | - | - | - | - | - |
| STAIR+NET high insecurity | 1.32 | 5.05 | 154.12 | 0.19 | .851 | -12.61 | 15.25 |
| Time x condition x insecurity status | |  |  |  |  |  |  |
| Mid-treatment SPS+NET low insecurity | - | - | - | - | - | - | - |
| Mid-treatment SPS+NET high insecurity | - | - | - | - | - | - | - |
| Mid-treatment STAIR+NET low insecurity | - | - | - | - | - | - | - |
| Mid-treatment STAIR+NET high insecurity | -10.72 | 8.18 | 149.12 | -1.31 | .192 | -26.88 | 5.45 |
| Post-treatment SPS+NET low insecurity | - | - | - | - | - | - | - |
| Post-treatment SPS+NET high insecurity | - | - | - | - | - | - | - |
| Post-treatment STAIR+NET low insecurity | - | - | - | - | - | - | - |
| Post-treatment STAIR+NET high insecurity | -22.94 | 8.57 | 152.57 | -2.68 | .008 | -39.87 | -6.00 |
| 3-month follow-up SPS+NET low insecurity | - | - | - | - | - | - | - |
| 3-month follow-up SPS+NET high insecurity | - | - | - | - | - | - | - |
| 3-month follow-up STAIR+NET low insecurity | - | - | - | - | - | - | - |
| 3-month follow-up STAIR + NET high insecurity | -24.35 | 9.10 | 155.10 | -2.68 | .008 | -42.32 | -6.37 |
| Baseline PCL score | 0.84 | 0.07 | 62.17 | 12.10 | < .001 | 0.71 | 0.98 |
|  |  |  |  |  |  |  |  |
| BDI score |  |  |  |  |  |  |  |
| Intercept | 46.51 | 2.45 | 112.56 | 18.96 | < .001 | 41.65 | 51.37 |
| Time |  |  |  |  |  |  |  |
| Pre-treatment | - | - | - | - | - | - | - |
| Mid-treatment | -2.61 | 2.50 | 153.50 | -1.04 | .298 | -7.54 | 2.33 |
| Post-treatment | -5.01 | 2.45 | 152.13 | -2.05 | .042 | -9.85 | -0.17 |
| 3-month follow-up | -4.95 | 2.50 | 153.51 | -1.98 | .049 | -9.89 | -0.02 |
| Condition |  |  |  |  |  |  |  |
| SPS+NET | - | - | - | - | - | - | - |
| STAIR+NET | 0.46 | 2.56 | 168.22 | 0.18 | .859 | -4.59 | 5.50 |
| Time x condition |  |  |  |  |  |  |  |
| Mid-treatment SPS+NET | - | - | - | - | - | - | - |
| Mid-treatment STAIR+NET | 1.18 | 3.36 | 150.14 | 0.53 | .596 | -4.85 | 8.42 |
| Post-treatment SPS+NET | - | - | - | - | - | - | - |
| Post-treatment STAIR+NET | 2.25 | 3.42 | 151.44 | 0.66 | .512 | -4.51 | 9.01 |
| 3-month follow-up SPS+NET | - | - | - | - | - | - | - |
| 3-month follow-up - STAIR+NET | 0.83 | 3.49 | 152.71 | 0.24 | .812 | -6.06 | 7.72 |
| Insecurity status |  |  |  |  |  |  |  |
| Low insecurity | - | - | - | - | - | - | - |
| High insecurity | 1.58 | 3.60 | 164.64 | 0.44 | .662 | -5.53 | 8.69 |
| Time x insecurity status |  |  |  |  |  |  |  |
| Mid-treatment low insecurity | - | - | - | - | - | - | - |
| Mid-treatment high insecurity | -3.69 | 4.56 | 149.38 | -0.81 | .420 | -12.70 | 5.32 |
| Post-treatment low insecurity | - | - | - | - | - | - | - |
| Post-treatment high insecurity | 4.06 | 4.68 | 151.43 | 0.87 | .387 | -5.18 | 13.30 |
| 3-month follow-up low insecurity | - | - | - | - | - | - | - |
| 3-month follow-up high insecurity | 12.36 | 4.88 | 153.58 | 2.53 | .012 | 2.72 | 22.00 |
| Condition x insecurity |  |  |  |  |  |  |  |
| SPS+NET low insecurity | - | - | - | - | - | - | - |
| SPS+NET high insecurity | - | - | - | - | - | - | - |
| STAIR+NET low insecurity | - | - | - | - | - | - | - |
| STAIR+NET high insecurity | 0.34 | 5.44 | 168.83 | 0.06 | .950 | -10.39 | 11.07 |
| Time x condition x insecurity status | |  |  |  |  |  |  |
| Mid-treatment SPS+NET low insecurity | - | - | - | - | - | - | - |
| Mid-treatment SPS+NET high insecurity | - | - | - | - | - | - | - |
| Mid-treatment STAIR+NET low insecurity | - | - | - | - | - | - | - |
| Mid-treatment STAIR+NET high insecurity | -3.32 | 6.79 | 145.78 | -0.49 | .625 | -16.73 | 10.09 |
| Post-treatment SPS+NET low insecurity | - | - | - | - | - | - | - |
| Post-treatment SPS+NET high insecurity | - | - | - | - | - | - | - |
| Post-treatment STAIR+NET low insecurity | - | - | - | - | - | - | - |
| Post-treatment STAIR+NET high insecurity | -9.18 | 7.09 | 149.43 | -1.29 | .197 | -23.19 | 4.83 |
| 3-month follow-up SPS+NET low insecurity | - | - | - | - | - | - | - |
| 3-month follow-up SPS+NET high insecurity | - | - | - | - | - | - | - |
| 3-month follow-up STAIR+NET low insecurity | - | - | - | - | - | - | - |
| 3-month follow-up STAIR + NET high insecurity | -15.02 | 7.48 | 152.40 | -2.01 | .046 | -29.80 | -0.25 |
| Baseline BDI score | 0.84 | 0.07 | 64.84 | 12.20 | < .001 | 0.70 | 0.98 |
|  |  |  |  |  |  |  |  |
| DSO – Emotion dysregulation |  |  |  |  |  |  |  |
| Intercept | 4.80 | 0.35 | 136.77 | 13.79 | < .001 | 4.11 | 5.48 |
| Time |  |  |  |  |  |  |  |
| Pre-treatment | - | - | - | - | - | - | - |
| Mid-treatment | -0.76 | 0.41 | 137.71 | -1.86 | .065 | -1.57 | 0.05 |
| Post-treatment | -1.26 | 0.43 | 139.09 | -2.96 | .004 | -2.10 | -0.42 |
| 3-month follow-up | -0.99 | 0.43 | 140.05 | -2.29 | .023 | -1.85 | -0.14 |
| Condition |  |  |  |  |  |  |  |
| SPS+NET | - | - | - | - | - | - | - |
| STAIR+NET | 0.12 | 0.48 | 136.73 | 0.25 | .801 | -0.82 | 1.07 |
| Time x condition |  |  |  |  |  |  |  |
| Mid-treatment SPS+NET | - | - | - | - | - | - | - |
| Mid-treatment STAIR+NET | 0.63 | 0.57 | 139.06 | 0.55 | .585 | -0.83 | 1.47 |
| Post-treatment SPS+NET | - | - | - | - | - | - | - |
| Post-treatment STAIR+NET | 0.32 | 0.58 | 139.06 | 0.55 | .585 | -0.83 | 1.47 |
| 3-month follow-up SPS+NET | - | - | - | - | - | - | - |
| 3-month follow-up - STAIR+NET | -0.12 | 0.60 | 140.71 | -0.20 | .840 | -1.32 | 1.07 |
| Insecurity status |  |  |  |  |  |  |  |
| Low insecurity | - | - | - | - | - | - | - |
| High insecurity | 0.03 | 0.65 | 137.48 | 0.05 | .959 | -1.25 | 1.31 |
| Time x insecurity status |  |  |  |  |  |  |  |
| Mid-treatment low insecurity | - | - | - | - | - | - | - |
| Mid-treatment high insecurity | 1.47 | 0.83 | 140.75 | 1.77 | .079 | -0.17 | 3.10 |
| Post-treatment low insecurity | - | - | - | - | - | - | - |
| Post-treatment high insecurity | 1.48 | 0.79 | 139.41 | 1.86 | .064 | -0.09 | 3.04 |
| 3-month follow-up low insecurity | - | - | - | - | - | - | - |
| 3-month follow-up high insecurity | 1.47 | 0.83 | 140.75 | 1.77 | .079 | -0.17 | 3.10 |
| Condition x insecurity |  |  |  |  |  |  |  |
| SPS+NET low insecurity | - | - | - | - | - | - | - |
| SPS+NET high insecurity | - | - | - | - | - | - | - |
| STAIR+NET low insecurity | - | - | - | - | - | - | - |
| STAIR+NET high insecurity | 0.31 | 1.11 | 136.57 | 0.28 | .782 | -1.88 | 2.49 |
| Time x condition x insecurity status | |  |  |  |  |  |  |
| Mid-treatment SPS+NET low insecurity | - | - | - | - | - | - | - |
| Mid-treatment SPS+NET high insecurity | - | - | - | - | - | - | - |
| Mid-treatment STAIR+NET low insecurity |  |  |  |  |  |  |  |
| Mid-treatment STAIR+NET high insecurity | -3.01 | 1.33 | 141.03 | -2.26 | .025 | -5.64 | -0.38 |
| Post-treatment SPS+NET low insecurity | - | - | - | - | - | - | - |
| Post-treatment SPS+NET high insecurity | - | - | - | - | - | - | - |
| Post-treatment STAIR+NET low insecurity | - | - | - | - | - | - | - |
| Post-treatment STAIR+NET high insecurity | -4.37 | 1.48 | 145.82 | -3.16 | .092 | -7.59 | -1.73 |
| 3-month follow-up SPS+NET low insecurity | - | - | - | - | - | - | - |
| 3-month follow-up SPS+NET high insecurity | - | - | - | - | - | - | - |
| 3-month follow-up STAIR+NET low insecurity | - | - | - | - | - | - | - |
| 3-month follow-up STAIR + NET high insecurity | -3.48 | 1.50 | 146.26 | -2.32 | .022 | -6.46 | -0.52 |
| Baseline emotion dysregulation score | 0.77 | 0.08 | 63.91 | 9.10 | < .001 | 0.60 | 0.94 |
|  |  |  |  |  |  |  |  |
| ITQ – Relationship Difficulties |  |  |  |  |  |  |  |
| Intercept | 4.51 | 0.35 | 142.31 | 12.98 | <.001 | 3.82 | 5.19 |
| Time |  |  |  |  |  |  |  |
| Pre-treatment | - | - | - | - | - | - | - |
| Mid-treatment | -0.73 | 0.45 | 139.86 | -1.62 | .107 | -1.62 | 0.16 |
| Post-treatment | -1.00 | 0.44 | 139.11 | -2.27 | .024 | -1.87 | -0.13 |
| 3-month follow-up | -0.74 | 0.42 | 137.27 | -1.75 | .083 | -1.58 | 0.099 |
| Condition |  |  |  |  |  |  |  |
| SPS+NET | - | - | - | - | - | - | - |
| STAIR+NET | 0.09 | 0.48 | 142.31 | 0.20 | .844 | -0.86 | 1.05 |
| Time x condition |  |  |  |  |  |  |  |
| Mid-treatment SPS+NET | - | - | - | - | - | - | - |
| Mid-treatment STAIR+NET | 0.17 | 0.59 | 137.94 | 0.30 | .768 | -1.00 | 1.35 |
| Post-treatment SPS+NET | - | - | - | - | - | - | - |
| Post-treatment STAIR+NET | -0.12 | 0.60 | 138.91 | -0.19 | .847 | -1.31 | 1.08 |
| 3-month follow-up SPS+NET | - | - | - | - | - | - | - |
| 3-month follow-up - STAIR+NET | -0.13 | 0.63 | 140.49 | -0.21 | .832 | -1.37 | 1.10 |
| Insecurity status |  |  |  |  |  |  |  |
| Low insecurity | - | - | - | - | - | - | - |
| High insecurity | -0.02 | 0.65 | 142.66 | -0.03 | .975 | -1.31 | 1.27 |
| Time x insecurity status |  |  |  |  |  |  |  |
| Mid-treatment low insecurity | - | - | - | - | - | - | - |
| Mid-treatment high insecurity | 0.83 | 0.79 | 136.50 | 1.05 | .294 | -0.73 | 2.39 |
| Post-treatment low insecurity | - | - | - | - | - | - | - |
| Post-treatment high insecurity | 1.69 | 0.82 | 139.06 | 2.06 | .041 | 0.07 | 3.32 |
| 3-month follow-up low insecurity | - | - | - | - | - | - | - |
| 3-month follow-up high insecurity | -0.02 | 0.65 | 142.66 | -0.03 | .975 | -1.31 | 1.27 |
| Condition x insecurity |  |  |  |  |  |  |  |
| SPS+NET low insecurity | - | - | - | - | - | - | - |
| SPS+NET high insecurity | - | - | - | - | - | - | - |
| STAIR+NET low insecurity | - | - | - | - | - | - | - |
| STAIR+NET high insecurity | 0.42 | 1.12 | 141.36 | 0.37 | .709 | -1.79 | 2.62 |
| Time x condition x insecurity status | |  |  |  |  |  |  |
| Mid-treatment SPS+NET low insecurity | - | - | - | - | - | - | - |
| Mid-treatment SPS+NET high insecurity | - | - | - | - | - | - | - |
| Mid-treatment STAIR+NET low insecurity | - | - | - | - | - | - | - |
| Mid-treatment STAIR+NET high insecurity | -1.92 | 1.38 | 140.55 | -1.39 | .167 | -4.65 | 0.81 |
| Post-treatment SPS+NET low insecurity | - | - | - | - | - | - | - |
| Post-treatment SPS+NET high insecurity | - | - | - | - | - | - | - |
| Post-treatment STAIR+NET low insecurity | - | - | - | - | - | - | - |
| Post-treatment STAIR+NET high insecurity | -2.59 | 1.53 | 145.98 | -1.69 | .093 | -5.61 | 0.44 |
| 3-month follow-up SPS+NET low insecurity | - | - | - | - | - | - | - |
| 3-month follow-up SPS+NET high insecurity | - | - | - | - | - | - | - |
| 3-month follow-up STAIR+NET low insecurity | - | - | - | - | - | - | - |
| 3-month follow-up STAIR + NET high insecurity | -3.31 | 1.56 | 146.40 | -2.13 | .035 | -6.38 | -0.23 |
| Baseline relationship difficulties score | 0.75 | 0.07 | 59.21 | 10.02 | <.001 | 0.60 | 0.90 |
|  |  |  |  |  |  |  |  |
| WHOQOL – Environmental | | |  |  |  |  |  |
| Intercept | 10.80 | 0.32 | 174.06 | 33.79 | < .001 | 10.16 | 11.43 |
| Time |  |  |  |  |  |  |  |
| Pre-treatment | - | - | - | - | - | - | - |
| Mid-treatment | 0.49 | 0.44 | 152.37 | 1.11 | .267 | -0.38 | 1.36 |
| Post-treatment | 1.49 | 0.44 | 152.15 | 3.37 | < .001 | 0.62 | 2.36 |
| 3-month follow-up | 1.47 | 0.45 | 153.61 | 3.28 | < .001 | 0.59 | 2.36 |
| Condition |  |  |  |  |  |  |  |
| SPS+NET | - | - | - | - | - | - | - |
| STAIR+NET | 0.08 | 0.45 | 173.78 | 0.17 | .868 | -0.82 | 0.97 |
| Time x condition |  |  |  |  |  |  |  |
| Mid-treatment SPS+NET | - | - | - | - | - | - | - |
| Mid-treatment STAIR+NET | -0.10 | 0.60 | 149.06 | -0.17 | .864 | -1.30 | 1.09 |
| Post-treatment SPS+NET | - | - | - | - | - | - | - |
| Post-treatment STAIR+NET | -1.09 | 0.61 | 149.70 | -1.79 | .075 | -2.29 | 0.11 |
| 3-month follow-up SPS+NET | - | - | - | - | - | - | - |
| 3-month follow-up - STAIR+NET | -0.73 | 0.63 | 152.71 | -1.14 | .254 | -1.98 | 0.53 |
| Insecurity status |  |  |  |  |  |  |  |
| Low insecurity | - | - | - | - | - | - | - |
| High insecurity | 0.01 | 0.62 | 174.08 | 0.01 | .991 | -1.22 | 1.23 |
| Time x insecurity status |  |  |  |  |  |  |  |
| Mid-treatment low insecurity | - | - | - | - | - | - | - |
| Mid-treatment high insecurity | -0.66 | 0.81 | 148.66 | -0.82 | .416 | -2.28 | 0.95 |
| Post-treatment low insecurity | - | - | - | - | - | - | - |
| Post-treatment high insecurity | -1.38 | 0.84 | 151.31 | -1.64 | .103 | -3.04 | 0.28 |
| 3-month follow-up low insecurity | - | - | - | - | - | - | - |
| 3-month follow-up high insecurity | -2.99 | 0.92 | 156.10 | -3.26 | < .001 | -4.81 | -1.18 |
| Condition x insecurity |  |  |  |  |  |  |  |
| SPS+NET low insecurity | - | - | - | - | - | - | - |
| SPS+NET high insecurity | - | - | - | - | - | - | - |
| STAIR+NET low insecurity | - | - | - | - | - | - | - |
| STAIR+NET high insecurity | -0.27 | 0.96 | 173.19 | -0.29 | .776 | -2.17 | 1.62 |
| Time x condition x insecurity status | |  |  |  |  |  |  |
| Mid-treatment SPS+NET low insecurity | - | - | - | - | - | - | - |
| Mid-treatment SPS+NET high insecurity | - | - | - | - | - | - | - |
| Mid-treatment STAIR+NET low insecurity | - | - | - | - | - | - | - |
| Mid-treatment STAIR+NET high insecurity | 1.78 | 1.22 | 145.14 | 1.46 | .147 | -0.63 | 4.19 |
| Post-treatment SPS+NET low insecurity | - | - | - | - | - | - | - |
| Post-treatment SPS+NET high insecurity | - | - | - | - | - | - | - |
| Post-treatment STAIR+NET low insecurity | - | - | - | - | - | - | - |
| Post-treatment STAIR+NET high insecurity | 2.13 | 1.27 | 148.96 | 1.68 | .095 | -0.38 | 4.64 |
| 3-month follow-up SPS+NET low insecurity | - | - | - | - | - | - | - |
| 3-month follow-up SPS+NET high insecurity | - | - | - | - | - | - | - |
| 3-month follow-up STAIR+NET low insecurity | - | - | - | - | - | - | - |
| 3-month follow-up STAIR + NET high insecurity | 3.24 | 1.38 | 153.65 | 2.35 | 0.02 | 0.52 | 5.95 |
| Baseline environmental quality of life score | 0.82 | 0.06 | 69.08 | 14.58 | <.001 | 0.71 | 0.93 |

CAPS = Clinician Administered PTSD Scale, PCL = PTSD Checklist for DSM-5, BDI= Beck Depression Inventory, ITQ = International Trauma Questionnaire, WHOQOL = World Health Organization Quality of Life Scale, CI = Confidence Interval
